# Keratin 6A promotes skin inflammation through JAK1-STAT3 activation in keratinocytes

**DOI:** 10.1186/s12929-025-01143-9

**Published:** 2025-05-09

**Authors:** Mengting Chen, Yaling Wang, Mei Wang, San Xu, Zixin Tan, Yisheng Cai, Xin Xiao, Ben Wang, Zhili Deng, Ji Li

**Affiliations:** 1 Department of Dermatology, Xiangya Hospital, Central South University, Changsha, China; 2 Hunan Key Laboratory of Aging Biology, Xiangya Hospital, Central South University, Changsha, China; 3 National Clinical Research Center for Geriatric Disorders, Xiangya Hospital, Central South University, Changsha, China; 4 Department of Dermatology, Hunan Provincial People’s Hospital, The First Affiliated Hospital of Hunan Normal University, Changsha, China

**Keywords:** Keratin 6, Rosacea, Psoriasis, Inflammation, JAK1-STAT3

## Abstract

**Background:**

Skin barrier dysfunction and immune activation are hallmarks of inflammatory skin diseases such as rosacea and psoriasis, suggesting shared pathogenic mechanisms. While barrier disruption may trigger or exacerbate skin inflammation, the precise underlying mechanisms remain unclear. Notably, epidermal barrier compromise leads to a marked increase in barrier alarmin expression. Among these, keratin 6A (KRT6A) plays a role in maintaining skin barrier integrity.

**Methods:**

We treated mouse skin and human keratinocytes, with and without KRT6A expression, with LL37/TNF-α and assessed the severity of inflammation. The specific mechanism by which KRT6A promotes skin inflammation was investigated using mass spectrometry and immunoprecipitation assays.

**Results:**

KRT6A expression was elevated in lesional skin from patients and mouse models of rosacea and psoriasis. In mice with LL37-induced rosacea-like and imiquimod (IMQ)-induced psoriasis-like skin inflammation, KRT6A knockdown alleviated inflammation, whereas KRT6A overexpression exacerbated inflammatory responses. Mechanistically, KRT6A activated signal transducer and activator of transcription 3 (STAT3) and enhanced proinflammatory cytokine expression in keratinocytes by reducing Janus kinase 1 (JAK1) ubiquitination. This occurred through inhibition of ring finger protein 41 (RNF41)-mediated JAK1 binding.

**Conclusions:**

Our findings indicate that KRT6A expression increases following epidermal barrier disruption and contributes to exacerbated skin inflammation in disease conditions. Targeting KRT6A may represent a novel therapeutic approach for inflammatory skin diseases associated with epidermal dysfunction.

**Supplementary Information:**

The online version contains supplementary material available at 10.1186/s12929-025-01143-9.

## Background

The epidermal barrier is essential for protecting the skin from physical, chemical, and biological insults. Its disruption contributes to inflammatory skin diseases such as rosacea, psoriasis, and atopic dermatitis [1, 2]. Rosacea and psoriasis, affecting up to 5.46% and 1.99% of the population, respectively [3, 4], and share immune dysregulation characterized by heightened Th1- and Th17-driven inflammation, mediated by cytokines such as IFN-γ, TNF-α, IL-17, and IL-22 [1, 2, 5–9]. While studies suggest that skin barrier dysfunction promotes immune responses by enhancing systemic allergic reactions and microbial penetration [10–12], its role in initiating inflammation in these diseases remains unclear.

The epidermal barrier comprises keratinocytes and intercellular domains [13]. Barrier disruption leads to aberrant keratin expression, as keratins are the primary structural proteins in keratinocytes [14]. Alarmins, a class of immune mediators released in response to tissue damage or infection, activate antigen-presenting cells and amplify immune responses [1, 15]. Skin barrier alarmins, including keratins 6 (KRT6), 16 (KRT16), and 17 (KRT17), are significantly upregulated following epidermal injury. Among KRT6 isoforms, KRT6A is the most predominant at the mRNA level in human skin and keratinocytes [16]. While KRT6 is normally expressed in glabrous skin, oral mucosa, and skin appendages—where it regulates epidermal proliferation, differentiation, and keratinocyte migration [17]—its role in epidermal dysfunction-related inflammation remains unclear.

Here, we investigate the role of KRT6A in skin inflammation. We demonstrate that KRT6A is significantly overexpressed in the epidermis of rosacea and psoriasis lesions. Functionally, KRT6A knockdown alleviates skin inflammation in mouse models of rosacea and psoriasis, whereas lentivirus-mediated KRT6A overexpression exacerbates inflammatory responses. Mechanistically, our findings reveal that KRT6A promotes JAK1-STAT3-mediated inflammation in keratinocytes by inhibiting the interaction between RNF41, an E3 ubiquitin ligase, and JAK1. Collectively, our study suggests that targeting KRT6A as a barrier alarmin may provide a novel therapeutic strategy for chronic inflammatory skin diseases.

## Materials and methods

### Datasets and analysis

We downloaded skin gene expression data of psoriasis lesions and healthy controls from the GEO database (GSE121212) or re-analyzed the previous rosacea data. Differentially expression analyses were conducted by unpaired Student's t-test in R. Proteins or genes with |log2 (foldchange)|> = 0.5&p.adj < 0.05 were identified as differentially expressed proteins (DEPs) or differentially expressed genes (DEGs). GO analysis of the DEPs and DEGs was performed by clusterProfiler R package [18].

### Reagents

The antimicrobial peptide LL37 was synthesized by Sangon Biotech with an amino acid sequence of LGDFFRKSKEKIGKEFKRIVQRIKDFLRNLVPRTES and a purity of > 95%. siKRT6A and siNC were synthesized by RiboBio (Guangzhou, China). The sequence of the probe is siNC: TTCTCCGAACGTGTCACGTdTdT, mouse-siKRT6A-1: CTCAGCTCTTCTACCATCA, mouse-siKRT6A-2: CACTGCTCATCTCTTTATA, human-siKRT6A-1: CAACAAGTTTGCCTCCTTCAT, human-siKRT6A-2: GAGGACTTCAAGAACAAATAT. MG132, cycloheximide (CHX), capsaicin, and puromycin were purchased from Selleck Chemical. Polybrene was purchased from Sigma-Aldrich. IL-23, IL-1β, IL6, IFN-γ, and TNFα were purchased from PeproTech. SPD304 was purchased from MCE.

### Lentivirus construction

The AAV-shKRT6A virus was constructed, identified, and provided by Hanbio Tech (China). The target sequence is CAACTTCTTGAGAGCTCTCTA. Full-length cDNA encoding human JAK1, KRT6A, RNF41, OBI1, TRIM21, and mouse KRT6A was amplified from the HEK293T or NIH3T3 cell cDNA library and subcloned into the vector plasmid. During the PCR, the sequence encoding the Flag tag or HA tag was added to the 5' end start codon of the coding DNA sequences (CDS) of the specified protein. The sequence of sh-JAK1 was obtained from the https://www.sigmaaldrich.cn/CN/zh and cloned into the plko.1 vector plasmid. The shRNA sequences targeting JAK1 mRNA were as follows: shJAK1# 1, 5′-GAGACTTCCATGTTACTGATT-3′ and shJAK1# 2, 5′-CTTGGCTACCTTGGAAACTTT-3′. Deletion of amino acids 134–317 yields RNF41 N-terminal mutant plasmid (RNF41-NT). Deletion of amino acids 1–133 yields RNF41 C-terminal mutant plasmid (RNF41-CT). The sequences targeting enzymatic-null RNF41 mutant mRNA were as follows: forward, 5′-CCTCATtctgaacaaGCTTTCTGCAACGCCTGC-3′ and reverse, 5′-GCttgttcagaATGAGGTGCCTGTACTGGCTCC-3′. All plasmids were used after successful sequencing by Tsingke Biotech. Lentiviruses were produced by co-transfecting constructed plasmids and the packaging plasmids pCMV-deltaR (ΔR) and pCMV-VSV-G (VSVG) into HEK293T cells using Fugene HD for 48 h to establish stable cell lines. Collect culture supernatants and concentrate with PEG-8000. The concentrated viral solution was added to the cell culture medium, and polybrene (10 μg/mL) was included to enhance infection efficiency. The culture medium was replaced 24 h after infection. After 72 h of infection, infected cells were screened with 1 μg/mL puromycin for 3 days. The vector plasmid, HA-Ub plasmid, ΔR, and VSVG plasmid were purchased from GenePharma.

### Human skin tissue samples

All human skin biopsies were performed at the Department of Dermatology in Xiangya Hospital, Central South University (Changsha, China). Human skins were taken from rosacea, psoriasis patients, or healthy individuals. This study was approved by the ethical committee of the Xiangya Hospital of Central South University (IRB number 202203076), and all subjects obtained written informed consent.

### Experimental animals and animal treatment

Eight-week-old female BALB/c mice were randomly assigned to different groups. To construct a rosacea-like mouse model, LL37 (320 umol) was injected intradermally on the back of mice at an interval of 12 h for two consecutive days (40 ul each time, for a total of 4 times). To construct a psoriasis-like mouse model, IMQ cream (25 mg, Aldara, 3 m Pharmaceuticals) is applied daily to the ears of mice for 6 days. To construct a mouse model with skin barrier dysfunction, tape stripping (Transpore TM, 3 M) was applied on the back of the mice at 12-h intervals for two consecutive days until the TEWL was elevated. To knock down or overexpress KRT6A locally in the skin of mice, adeno-associated virus, lentivirus, or siRNA should be injected intradermally into the back or ears of mice in advance as described in the procedure. As previously described, the redness area and redness score were measured 12 h after the last injection of LL37 [19].

### Histology and immunohistochemistry (IHC)

The skin tissue samples were fixed with formalin, embedded in paraffin, and cut into 3-μm sections for hematoxylin and eosin (H&E) and immunohistochemistry. Immunohistochemistry was performed as previously described [20]. Skin sections were incubated with anti-KRT6A antibody (Abcam) or anti-CD31 antibody (CST, 77699). Photographs were taken from three typical areas of each sample. Images were acquired using a Zeiss Axio Scope A1 microscope (Zeiss, Germany) and processed using Image J software (National Institutes of Health, Bethesda, MD).

### Cell culture and treatment

The HaCaT cell line, NIH3T3 cell line, and HEK293T cell line used in this study were derived from NTCC (Biovector Science Laboratory, Beijing, China). HaCaT cells were grown in a calcium-free DMEM medium containing 10% fetal bovine serum, penicillin–streptomycin, and 2 mM glutamine (Invitrogen). The original medium was replaced with DMEM containing 1.8 M calcium ions before drug treatment. For immunofluorescence, HaCaT cells were treated with TNFα (100 ng/ml) for 2 h. HaCaT cells were treated with IL6 (20 ng/ml), IFNr (50 ng/ml), TNFα (100 ng/ml), LL37, capsaicin (1 μM or 10 μM), or UVB (10 mJ/cm^2^ or 25 mJ/cm^2^, Opsytec Dr. Gröbel) for 24 h and used for western blotting. For heat shock treatment, HaCaT cells were treated with heat shock (37, 42, or 44 °C) in a circulating water bath for half an hour and rested for 24 h before being tested. For CHX treatment, HaCaT cells were treated with CHX (10 μM) for the indicated time before collection. We transfected siRNAs and plasmids using Lipofectamine RNAIMAX (Invitrogen) according to the manufacturer's instructions. HEK293T cells and NIH3T3 cells were grown in a DMEM medium (Gibco, Shanghai, China) containing 10% fetal bovine serum and penicillin–streptomycin. All experiments should be performed at least three times.

### RNA extraction, real-time quantitative PCR (RT-qPCR), and RNAseq

Total RNA was extracted from mouse skin tissue or HaCaT cells with TRIzol (Thermo Fisher Scientific). RNA (1 μg) was reverse transcribed to cDNA using the PrimeScript™ RT reagent Kit with gDNA Eraser (RR047A Takara). RT-qPCR was performed with ChamQ Universal SYBR qPCR Master Mix (Vazyme, Q711-02) using a LightCycler 96 thermocycler (Roche, Basel, Switzerland). Taking the expression of GAPDH as a reference, the relative expression levels of each gene were evaluated by the delta CT method, and the fold changes were normalized to the control group. The specific primer sequences are shown in Supplementary Table S1.

### Co-immunoprecipitation (Co-IP) assay and mass spectrometry (MS)

Flag-JAK1, KRT6A, HA-Ub, HA-RNF41, HA-RNF41-CT, or HA-RNF41-NT plasmids were transfected in HEK293T cells for 48 h. HEK293T were lysed with NP-40 Lysis Buffer (Beyotime, P0013) after 4 h of treatment with MG132 (50 uM) and then collected after 2-h incubation with anti-Flag magnetic beads (Selleck, B26102). Wash the beads 3 times with wash buffer (25 mM Tris (pH 8.0), 150 mM NaCl, 0.2% NP40) and then boil in SDS loading buffer. Immunoprecipitated protein complexes were detected by Western blotting or by mass spectrometry. The Ultimate 3000 RSLCnano system was used for separating peptides and the Q Exactive (Thermo Fischer Scientific, San Jose, CA, USA) was used for analysis. Proteome discoverer version 1.4 (PD1.4; Thermo Fisher Scientific) and the search algorithm Mascot were used for protein identification.

### Immunofluorescence (IF) analysis

Mouse skins were embedded with o.c.t (Tissue Tek) and then cut into 8-μm slices. Tissue slices and treated HaCaT cells are fixed in 4% paraformaldehyde and then blocked using a blocking solution (5% Normal Donkey Serum, 0.3% Triton X-100 in PBS) for 1 h at room temperature. Incubate with primary antibody overnight at 4 °C. Primary antibodies are shown in Supplementary Table S2. Sections were incubated with Alexa Fluor 488 or 594-conjugated secondary antibodies for 1 h at room temperature and then stained with 4′,6-diamidino-2-phenylindole (DAPI). All photos were taken using a Zeiss Axio Scope A1 microscope (Zeiss, Germany).

### Cytoplasmic and nuclear protein extraction

Cytoplasmic and nuclear proteins were separated according to the instructions of the Nuclear and Cytoplasmic Protein Extraction Kit (Beyotime, P0028). Briefly, cells are centrifuged at 800 × *g* at 4 °C for 10 min after washing with PBS. Add Cytoplasmic Protein Extraction Reagent A to the cell pellet, lyse on ice for 15 min, and then add Cytoplasmic Protein Extraction Reagent B for 1 min. Centrifuge at 12,000 × *g* at 4 °C for 5 min, and the supernatant is the cytoplasmic proteins. The nucleus is present in the pellet and resuspended in the Nuclear Protein Extraction Reagent. Vortex vigorously every 1–2 min for 15–30 s for a total of 30 min. Centrifuge at 12,000 × *g* at 4 °C for 10 min, and the supernatant is the nuclear proteins.

### Western Blotting

Proteins were separated by sodium dodecyl sulfate–polyacrylamide gel electrophoresis and transferred to PVDF membranes. Membranes were blocked with 5% skim milk for 1 h at room temperature and then incubated with primary antibodies overnight at 4 °C. Primary antibodies were shown in Table S2. The next day, PVDF membranes were incubated with HRP-conjugated secondary antibodies (ZSGB-BIO) and visualized on a ChemiDoc TM using HRP substrate (Luminata, Millipore) on the XRS + system (Bio-Rad). The expression levels of TUBULIN, HSP90, and GAPDH were used as controls.

### Protein–protein interaction prediction

The primary sequences of human JAK1 (UniProt ID: P23458) and KRT6A (UniProt ID: P02538) were obtained from UniProtKB [21]. A heterodimeric complex was predicted using ColabFold v1.5.5 [22], a cloud-based AlphaFold2 framework [23], with paired sequence inputs. The top-ranked model (selected by highest confidence scores) was analyzed using ChimeraX v1.9.

### Statistical analysis

GraphPad Prism 9 software was used for statistical analysis. Data represent the mean ± SEM. Normality analysis was performed on the data before comparison. The two-tailed unpaired Student's t-test was used for comparison between the two groups, and the one-way ANOVA with Bonferroni's posthoc tests was used for multiple comparisons. The p-value < 0.05 (expressed as *p < 0.05, **p < 0.01, ***p < 0.001, or no significant (NS)) was significant.

## Results

### KRT6A is upregulated in inflammatory skin diseases

**Fig. 1:**
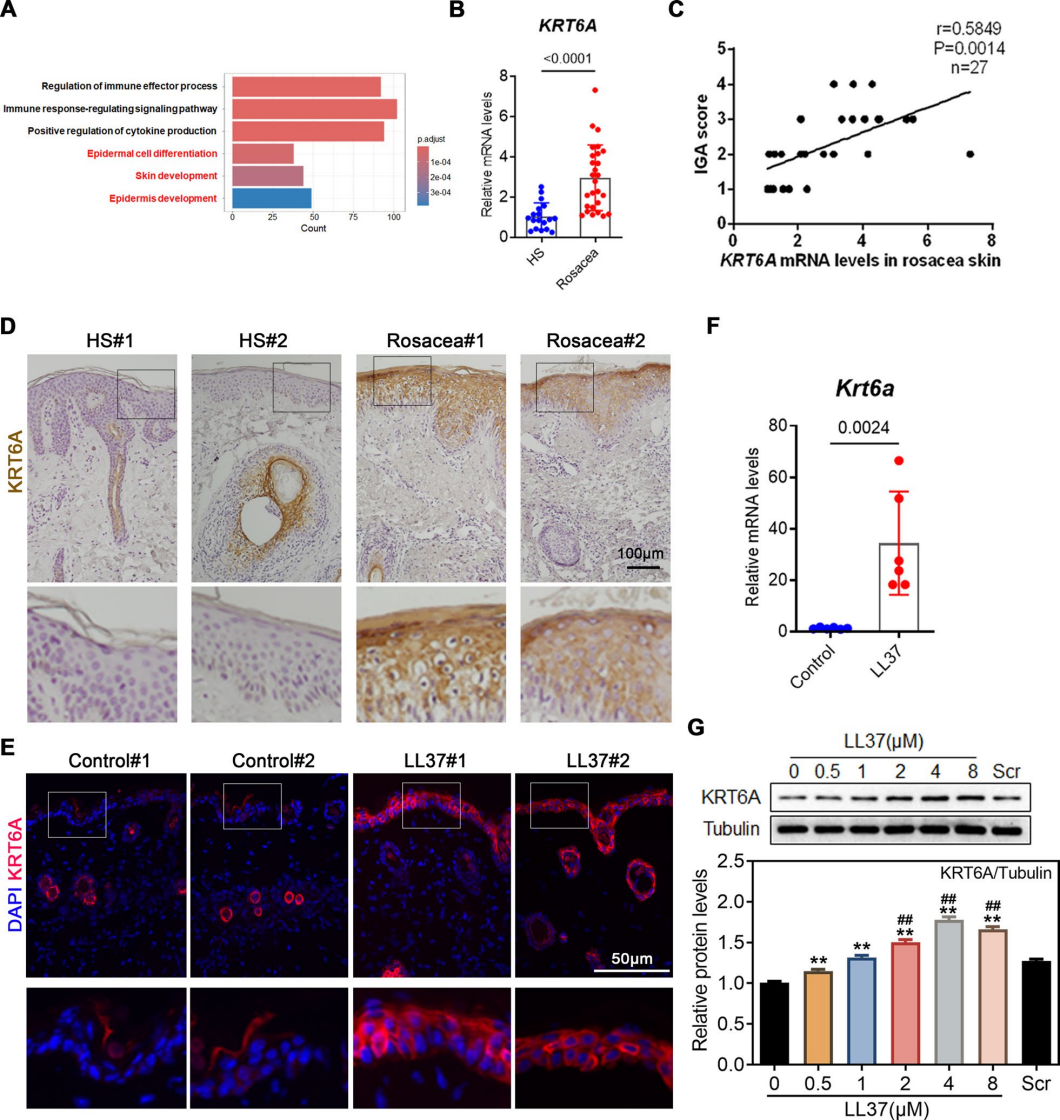
KRT6A is up-regulated in rosacea. **A** GO analysis showed that differentially expressed genes in rosacea lesions were associated with immune response and skin development. **B** The mRNA expression levels of KRT6A in the skin of HS (n = 19) and patients with rosacea (n = 46). **C** Correlation analysis between KRT6A expression and IGA score in rosacea lesions. **D** Representative immunohistochemistry (IHC) images showing the expression of KRT6A in rosacea lesions and healthy controls. **E** Representative immunofluorescence (IF) images showing the expression of KRT6A in LL37-induced rosacea-like mice. **F** RT-qPCR analysis showing the mRNA expression of KRT6A in the skin of LL37-induced rosacea-like mice and controls. **G** Representative western blot images showing the expression of KRT6A in LL37-treated keratinocytes. Quantification of relative protein expression is shown in the bottom panel. Scr: scrambled 37 amino acid polypeptide sequences. *P < 0.05; **P < 0.01, compare with the 0 group; #*P* < 0.05; ##*P* < 0.01, compare with the Scr group. All experiments were performed in 3 independent biological replicates. Data represents the mean ± SEM. Two-tailed unpaired Student’s t-test (**B**, **F**, **G**) was used

To investigate the role of the skin barrier in rosacea, we analyzed the gene expression profile of rosacea lesions using RNA sequencing (RNA-seq). DEGs were enriched not only in inflammation-related pathways, such as immune response regulation and cytokine production, but also in biological processes related to skin barrier integrity, including epidermal development, epidermal cell differentiation, and skin development (Fig. 1A). These findings suggest epithelial barrier alterations in rosacea. Given that skin barrier alarmins contribute to barrier composition and respond rapidly to stress, we next examined their expression in rosacea lesions. Compared with healthy controls, *KRT6A* and *KRT16* mRNA levels were significantly upregulated in rosacea, whereas *CLDN1*, *CLDN16*, *OCLN*, and *KRT17* showed no significant changes, and *CLDN23* was significantly downregulated (Supplementary Fig. 1A). Among these, KRT6A exhibited the pronounced increase. Given its upregulation in rosacea and in mice with barrier dysfunction, we focused on KRT6A in this study (Supplementary Fig. 1C).

We first analyzed KRT6A RNA levels and their clinical correlation in rosacea lesions. KRT6A expression was significantly increased in rosacea lesions and positively correlated with Investigator’s Global Assessment (IGA) scores but not with Clinical Erythema Assessment (CEA) scores (Fig. 1B, C and Supplementary Fig. 1B), indicating its involvement in inflammation. Immunohistochemistry (IHC) further revealed that KRT6A, normally localized to hair follicles in healthy skin, was markedly increased in the epidermis of rosacea patients (Fig. 1D). Since LL37 treatment is a well-established model for rosacea in vitro and in vivo [24], we examined KRT6A expression in LL37-induced rosacea-like mice. Both mRNA and protein levels of KRT6A were significantly upregulated, as confirmed by immunofluorescence (IF) and real-time quantitative PCR (RT-qPCR) (Fig. 1E, F). In HaCaT keratinocytes treated with LL37/TNF-α to mimic the in vitro disease environment, KRT6A protein expression was also elevated (Fig. 1G and Supplementary Fig. 1E). Additionally, known rosacea triggers—including capsaicin, heat stimulation, and UVB irradiation [25]—increased KRT6A expression in HaCaT cells (Supplementary Fig. 1D). To investigate how these stimuli regulate KRT6A expression, we first examined TNF-α levels following capsaicin, heat, and UVB exposure. All these stimuli induced TNF-α expression (Supplementary Fig. 1F). Furthermore, treatment with SPD304, a TNF-α inhibitor, effectively suppressed heat-induced TNF-α upregulation, suggesting that these stimuli regulate KRT6A expression at least partially through TNF-α signaling (Supplementary Fig. 1G).

KRT6A upregulation was also observed in psoriasis lesions and IMQ-induced psoriasis-like mouse models, where its expression was localized primarily to epidermal keratinocytes (Supplementary Fig. 2A–D). These results demonstrate that KRT6A is consistently upregulated in the epidermis of inflammatory skin diseases, including rosacea and psoriasis.

### KRT6A knockdown attenuates skin inflammation in rosacea and psoriasis

**Fig. 2:**
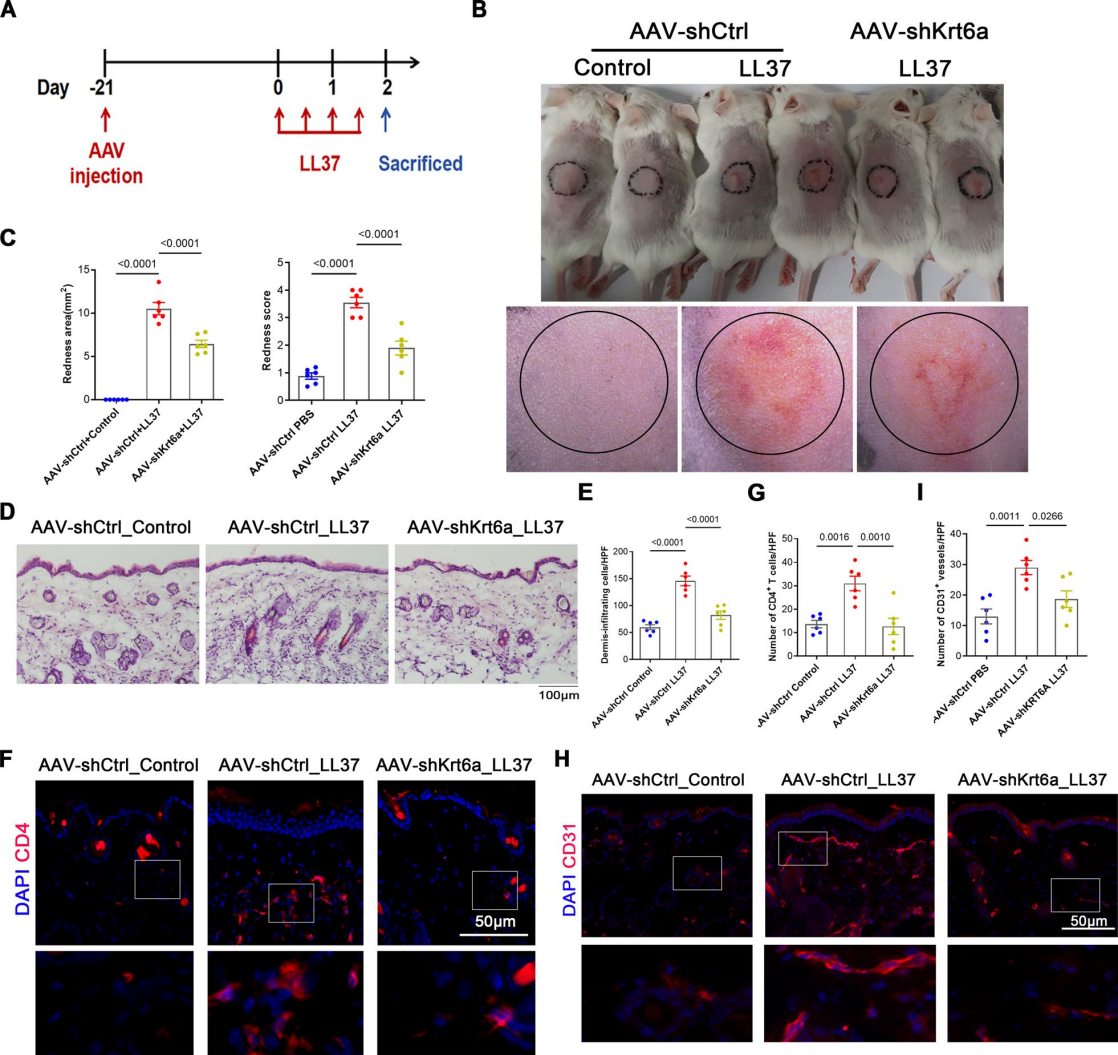
KRT6A knockdown relieves the development of rosacea. **A** Schematic diagram of AAV-shKRT6A-injected mice treated with LL37 or PBS. **B** The back skins of the control group and AAV shRNA-mediated knockdown of the Krt6a group treated with or without LL37 (n = 6/group). **C** The severity of the rosacea-like phenotype was evaluated on account of the redness area and score. **D** HE staining of lesional skin sections from (**B**). **E** Quantitative result of HE staining for dermal cellular infiltrates is shown. Data represent the mean ± SEM. **F** Immunostaining of CD4 in skin sections. **G** Quantitative result of CD4^+^ T cells is shown. Data represent the mean ± SEM. **H** Immunostaining of CD31 in skin sections. **G** Quantitative result of CD31^+^ vessels is shown. Data represent the mean ± SEM. 1-way ANOVA with Bonferroni’s post hoc test (**C**, **E**, **G**, **I**) was used

To investigate the role of KRT6A in rosacea, we designed a short hairpin RNA (shRNA) targeting KRT6A and packaged it into a recombinant adeno-associated virus serotype 9 (AAV-shKRT6A). Mice were injected with AAV-shKRT6A and subjected to LL37-induced rosacea-like inflammation (Fig. 2A). Immunofluorescence (IF) confirmed successful KRT6A knockdown in AAV-infected mice (Supplementary Fig. 3A). Compared with controls, KRT6A knockdown significantly alleviated the rosacea-like phenotype, as indicated by reduced erythema scores and affected areas (Fig. 2B, C). Histological analysis revealed a marked decrease in inflammatory cell infiltration in KRT6A-knockdown mice (Fig. 2D, E). Given that rosacea is characterized by inflammatory cell infiltration and vascular proliferation [26], we further assessed CD4^+^ T cells and CD31^+^ blood vessels in the skin. Both were significantly reduced in the KRT6A-knockdown group compared with controls (Fig. 2F–I).

To extend these findings to psoriasis, we evaluated the effects of KRT6A knockdown in an IMQ-induced psoriasis-like mouse model (Supplementary Fig. 4A). IF staining confirmed efficient knockdown of KRT6A (Supplementary Fig. 4B), which significantly improved psoriasis-like pathology, including acanthosis and inflammatory leukocyte infiltration (Supplementary Fig. 4C). Hematoxylin and eosin (H&E) staining further demonstrated reduced dermal immune cell infiltration and decreased epidermal thickness in KRT6A-knockdown mice (Supplementary Fig. 4D-E). Additionally, IF staining showed a significant reduction in CD4^+^ T cells and Ki67^+^ proliferating cells in KRT6A-knockdown skin (Supplementary Fig. 4F-I). Similar anti-inflammatory effects were observed following KRT6A knockdown via small interfering RNA (siRNA) in both LL37- and IMQ-induced models (Supplementary Fig. 3B-C and Supplementary Fig. 4 J-K). Collectively, these findings suggest that targeting epidermal KRT6A alleviates inflammation in rosacea and psoriasis, highlighting its potential as a therapeutic target for inflammatory skin diseases.

### KRT6A overexpression exacerbates skin inflammation in rosacea and psoriasis

**Fig. 3:**
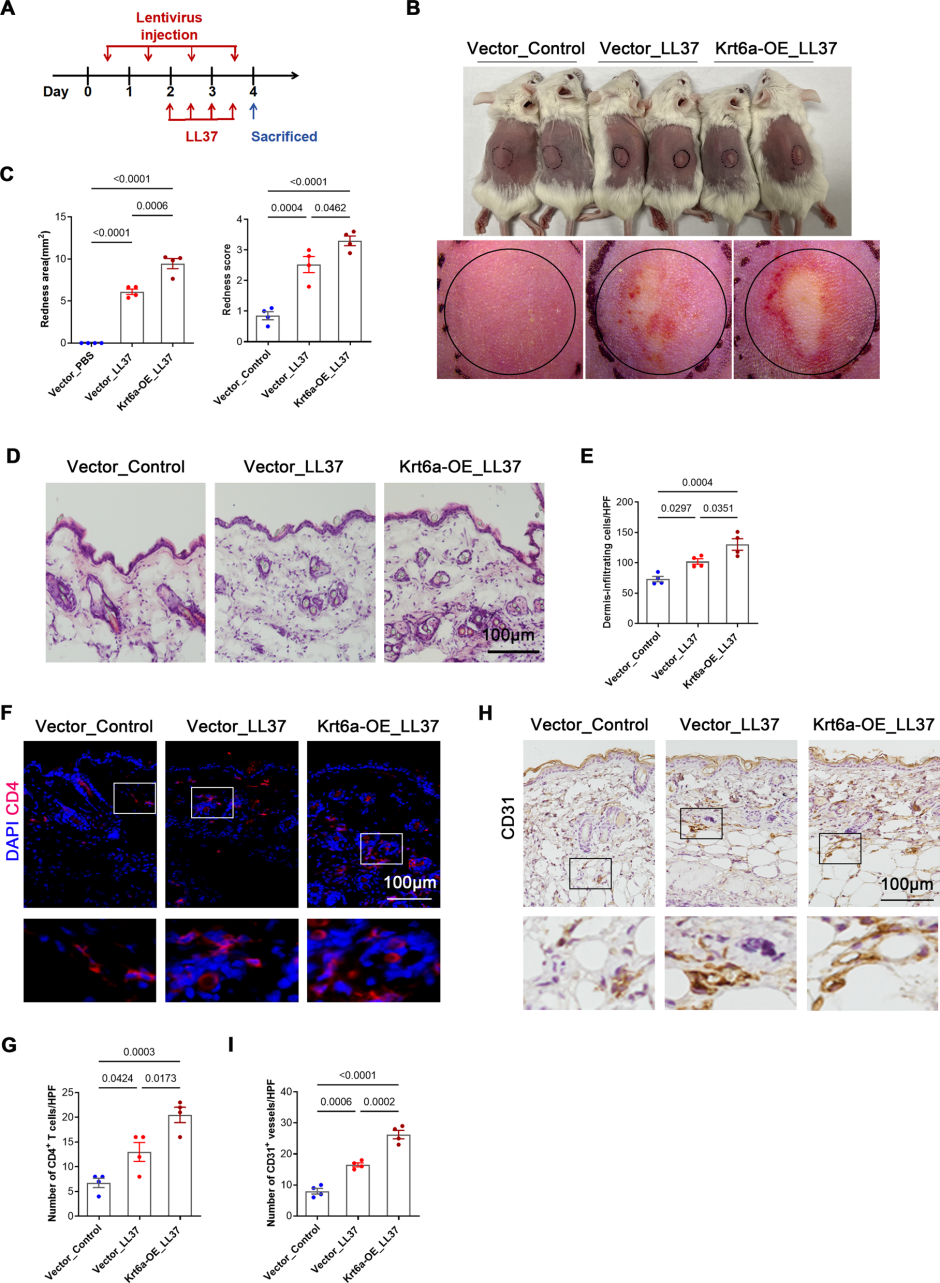
KRT6A aggravates skin inflammation in rosacea. **A** Schematic diagram of lentivirus-injected mice treated with LL37 or PBS. **B** The back skin of mice in control group and KRT6A-overexpressed group injected with LL37 or PBS (n = 4/group). **C** The severity of the rosacea-like phenotype was evaluated on account of the redness area and score. **D** HE staining of lesional skin sections from (**B**). **E** Quantitative result of HE staining for dermal cellular infiltrates is shown. Data represent the mean ± SEM. **F** Immunostaining of CD4 in skin sections. **G** Quantitative result of CD4^+^ T cells is shown. Data represent the mean ± SEM. **H** Immunostaining of CD31 in skin sections. **I** Quantitative result of CD31^+^ vessels is shown. Data represent the mean ± SEM. 1-way ANOVA with Bonferroni’s post hoc test (**C**, **E**, **G**, **I**) was used

To determine whether KRT6A contributes to skin inflammation, we performed intradermal injections of a lentivirus encoding KRT6A to induce its overexpression (Fig. 3A). Immunofluorescence (IF) confirmed successful overexpression in the epidermis (Supplementary Fig. 5A). Notably, KRT6A-overexpressing mice exhibited significantly higher erythema scores and larger erythematous areas compared with controls (Fig. 3B, C). Histological analysis revealed increased inflammatory cell infiltration following KRT6A overexpression (Fig. 3D, E). Additionally, IF staining demonstrated a significant increase in CD4^+^ T cell infiltration and CD31^+^ blood vessel density in the KRT6A-overexpression group (Fig. 3F–I).

To further validate these findings, we assessed the effects of KRT6A overexpression in IMQ-induced psoriasis-like mice (Supplementary Fig. 6A). Consistent with our observations in rosacea, KRT6A overexpression exacerbated psoriasis-like phenotypic and histological changes, including enhanced epidermal thickening and inflammatory cell infiltration (Supplementary Fig. 6B-E). Moreover, IF staining confirmed increased CD4^+^ T cell infiltration and a higher number of Ki67^+^ proliferating cells in KRT6A-overexpressing mice (Supplementary Fig. 6F–I).

Collectively, these results indicate that epidermal KRT6A promotes the pathogenesis of inflammatory skin diseases, including rosacea and psoriasis, highlighting its potential role as a therapeutic target.

### KRT6A promotes inflammation by regulating the activation of STAT3 in keratinocytes

**Fig. 4:**
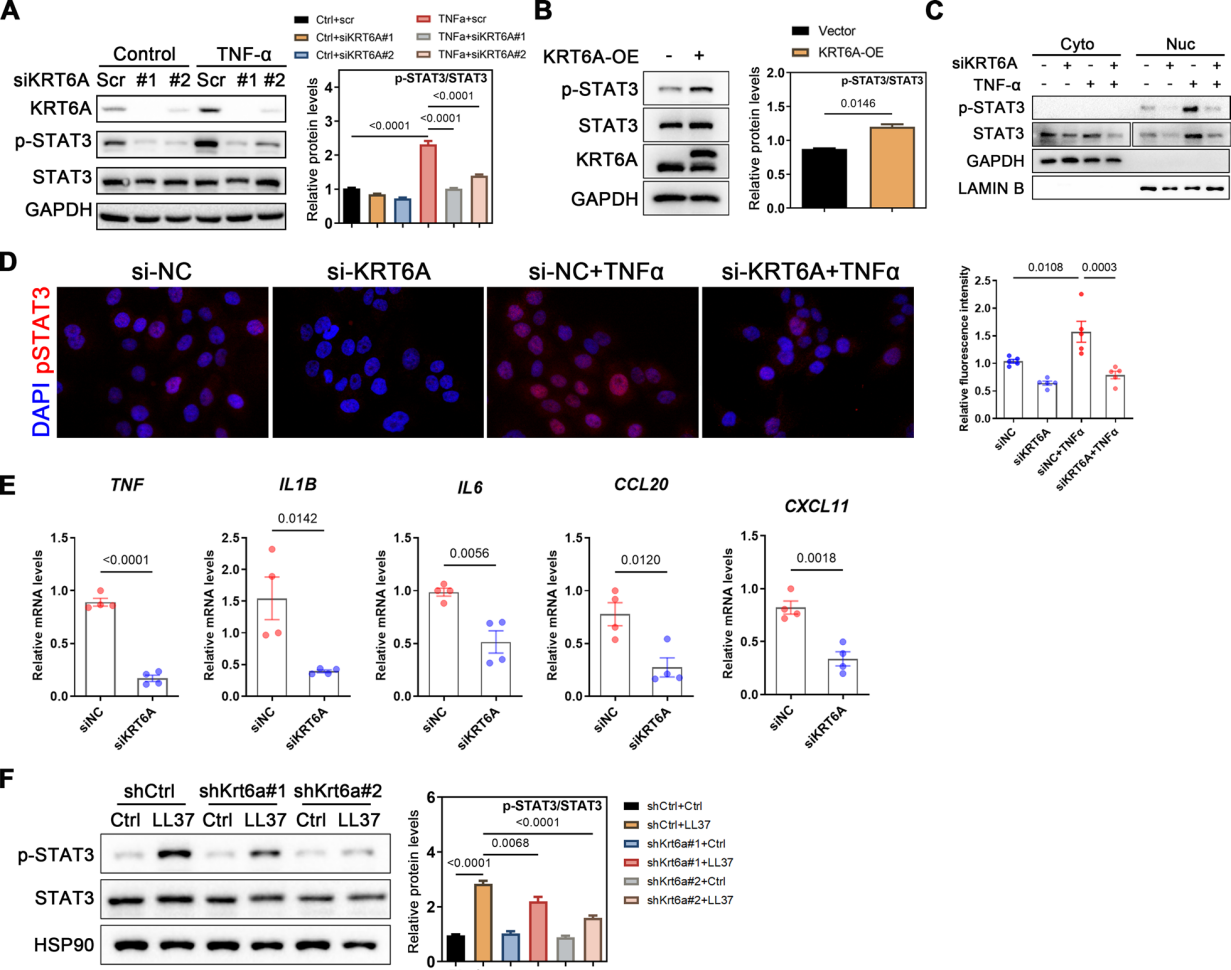
KRT6A affects STAT3 activation and downstream cytokine expression in keratinocytes. **A** Immunoblotting of p-STAT3, STAT3, p-STAT1, STAT1, p-p65, P65, p-p38, P38, p-AKT, AKT, p-ERK or ERK in cell lysates from HaCaT cells infected with siKRT6A or Scr and stimulated with TNF-α for 2 h. Quantification of relative protein expression is shown in the right panel. **B** The STAT3 activation in KRT6A-overexpressed HaCaT cells. Quantification of relative protein expression is shown in the right panel. **C** Expression of STAT3 and pSTAT3 in cytoplasm or nucleus from HaCaT cells infected with siKRT6A or Scr and stimulated with TNF-α for 2 h. **D** Expression (left) and relative fluorescence intensity (right) of pSTAT3 in KRT6A-knockdown HaCaT cells treated with or without TNF-α for 2 h. **E** The mRNA levels of downstream cytokines in KRT6A-knockdown HaCaT cells. **F** Immunoblotting of p-STAT3 and STAT3 in skin lesions from LL37-induced mice treated with AAV-scr or Krt6a shRNA. Quantification of relative protein expression is shown in the right panel. All experiments were performed in 3 independent biological replicates. Data represents the mean ± SEM. *P < 0.05; **P < 0.01, ns for not significant. Two-tailed unpaired Student’s t-test (**E**) or 1-way ANOVA with Bonferroni’s post hoc test (**A**, **B**, **D**, **F**) was used

Given that multiple signaling pathways contribute to the pathogenesis of rosacea and psoriasis [27–29], we investigated the downstream mechanisms of KRT6A by examining changes in these pathways following KRT6A knockdown via siRNA in HaCaT keratinocytes. Western blot analysis revealed that phosphorylated STAT3 (p-STAT3) was significantly upregulated upon TNF-α stimulation but was suppressed following KRT6A knockdown (Fig. 4A). Conversely, KRT6A overexpression enhanced STAT3 phosphorylation without significantly affecting p-Akt or p-Erk levels (Fig. 4B and Supplementary Fig. 7B). Since STAT3 exerts its effects through nuclear translocation upon phosphorylation, we further demonstrated that KRT6A knockdown reduced TNF-α-induced nuclear translocation of STAT3 and decreased nuclear p-STAT3 expression (Fig. 4C, D).

To assess the impact of KRT6A on STAT3 downstream cytokines, we performed RT-qPCR and found that KRT6A knockdown significantly downregulated key proinflammatory genes, including *IL1B*, *IL6*, *TNFα*, *CCL20*, and *CXCL11*, in HaCaT cells (Fig. 4E). Consistent with these findings, KRT6A downregulation also suppressed STAT3 phosphorylation in rosacea-like mouse lesions (Fig. 4F). To further validate the role of KRT6A in inflammation, we examined cytokine expression in LL37- and IMQ-induced mouse models following KRT6A knockdown or overexpression, with results mirroring those of the in vitro experiments (Supplementary Fig. 8A–D). Additionally, we explored the relationship between KRT6A and skin barrier integrity by assessing the expression of CLDN4 and CLDN23, two barrier-related molecules. KRT6A knockdown rescued the disease-associated downregulation of these molecules in rosacea and psoriasis mouse models (Supplementary Fig. 8E), suggesting that KRT6A may contribute to skin inflammation by modulating barrier function.

In conclusion, our findings indicate that KRT6A plays a pivotal role in skin inflammation, primarily by activating the STAT3 pathway and promoting the production of downstream proinflammatory cytokines in keratinocytes.

### KRT6A regulates STAT3 activation and downstream cytokines by targeting JAK1

**Fig. 5:**
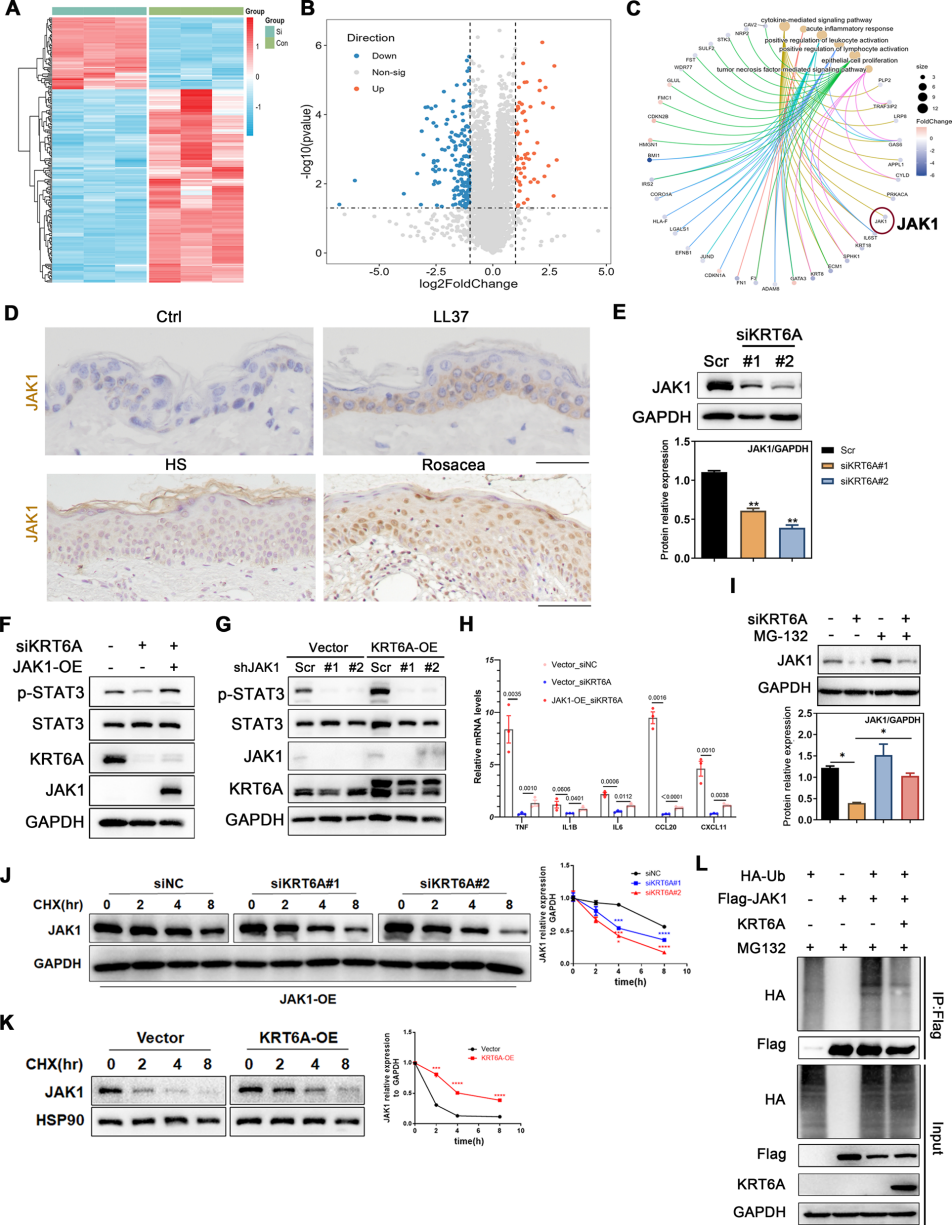
KRT6A increases JAK1 expression by targeting the ubiquitination. The heat map (**A**), volcano graph (**B**) and GO analysis (**C**) of differentially expressed protein in KRT6A-knockdown HaCaT cells. **D** Representative IHC images showing the expression of JAK1 in rosacea lesions and healthy controls in the upper panel; Representative IHC images showing the expression of JAK1 in LL37-induced rosacea-like mice in the bottom panel. **E** The JAK1 expression in KRT6A-knockdown HaCaT cells. Quantification of relative protein expression is shown in the bottom panel. **F** The STAT3 activation in KRT6A-knockdown HaCaT cells combined with or without JAK1 overexpression. **G** The STAT3 activation in KRT6A-overexpressed HaCaT cells combined with or without JAK1 knockdown. **H** The mRNA levels of downstream cytokine in KRT6A-knockdown HaCaT cells combined with or without JAK1 overexpression. **I** The JAK1 expression in KRT6A-knockdown HaCaT cells treated with or without MG132. Quantification of relative protein expression is shown in the bottom panel. **J** The JAK1 expression treated with CHX in control or KRT6A-knockdown HaCaT cells. Quantification of relative protein expression is shown in the right panel. **K** The JAK1 expression treated with CHX in control or KRT6A-overexpressed HaCaT cells. Quantification of relative protein expression is shown in the right panel. **L** The ubiquitination of JAK1 with or without KRT6A overexpression. All experiments were performed in 3 independent biological replicates. Data represents the mean ± SEM. *P < 0.05; **P < 0.01, ***P < 0.001, ****P < 0.0001. Two-tailed unpaired Student’s t-test (**E**) or 1-way ANOVA with Bonferroni’s post hoc test (**I**, **J**, **K**) was used

To elucidate how KRT6A regulates STAT3 activation in keratinocytes, we performed mass spectrometry to identify DEPs following KRT6A knockdown. The analysis revealed significant changes in protein expression, with DEPs enriched in inflammatory response and cytokine signaling pathways. Notably, JAK1, a key upstream regulator of STAT3, was among the affected proteins (Fig. 5A–C). To investigate the role of JAK1, we firstly examined its expression in rosacea and psoriasis patient samples as well as corresponding mouse models. Our findings demonstrated a significant upregulation of JAK1 in the epidermis (Fig. 5D and Supplementary Fig. 9A). Functional experiments further confirmed that KRT6A knockdown significantly reduced JAK1 protein levels (Fig. 5E), while JAK1 overexpression rescued STAT3 phosphorylation in KRT6A-knockdown cells (Fig. 5F). Conversely, JAK1 knockdown attenuated the increase in STAT3 phosphorylation induced by KRT6A overexpression (Fig. 5G). Additionally, RT-qPCR analysis showed that JAK1 overexpression reversed the downregulation of key inflammatory cytokines (*IL1B*, *IL6*, *TNFα*, *CCL20*, *CXCL11*) caused by KRT6A knockdown (Fig. 5H). These findings establish that KRT6A regulates STAT3 phosphorylation and downstream cytokine production via JAK1.

Protein degradation plays a crucial role in regulating JAK-STAT signaling, with previous studies implicating the ubiquitin–proteasome system in JAK1 turnover [30, 31]. To explore whether KRT6A influences JAK1 stability through this pathway, we treated HaCaT cells with MG132, a proteasome inhibitor. MG132 treatment rescued KRT6A knockdown-induced JAK1 downregulation (Fig. 5I), suggesting that KRT6A suppresses JAK1 degradation. Protein degradation assays further demonstrated that KRT6A knockdown accelerated JAK1 degradation, while KRT6A overexpression promoted JAK1 accumulation (Fig. 5J, K). To determine whether KRT6A regulates JAK1 stability via ubiquitination, we assessed JAK1 ubiquitination in HEK293T cells. Overexpression of KRT6A significantly reduced JAK1 ubiquitination (Fig. 5L), confirming that KRT6A inhibits JAK1 degradation by suppressing its ubiquitination.

Overall, these findings demonstrate that KRT6A stabilizes JAK1 by inhibiting its ubiquitination-mediated degradation, thereby promoting JAK1-STAT3 signaling and inflammatory cytokine production in keratinocytes.

### KRT6A regulates JAK1 ubiquitination by limiting its interaction with RNF41

**Fig. 6:**
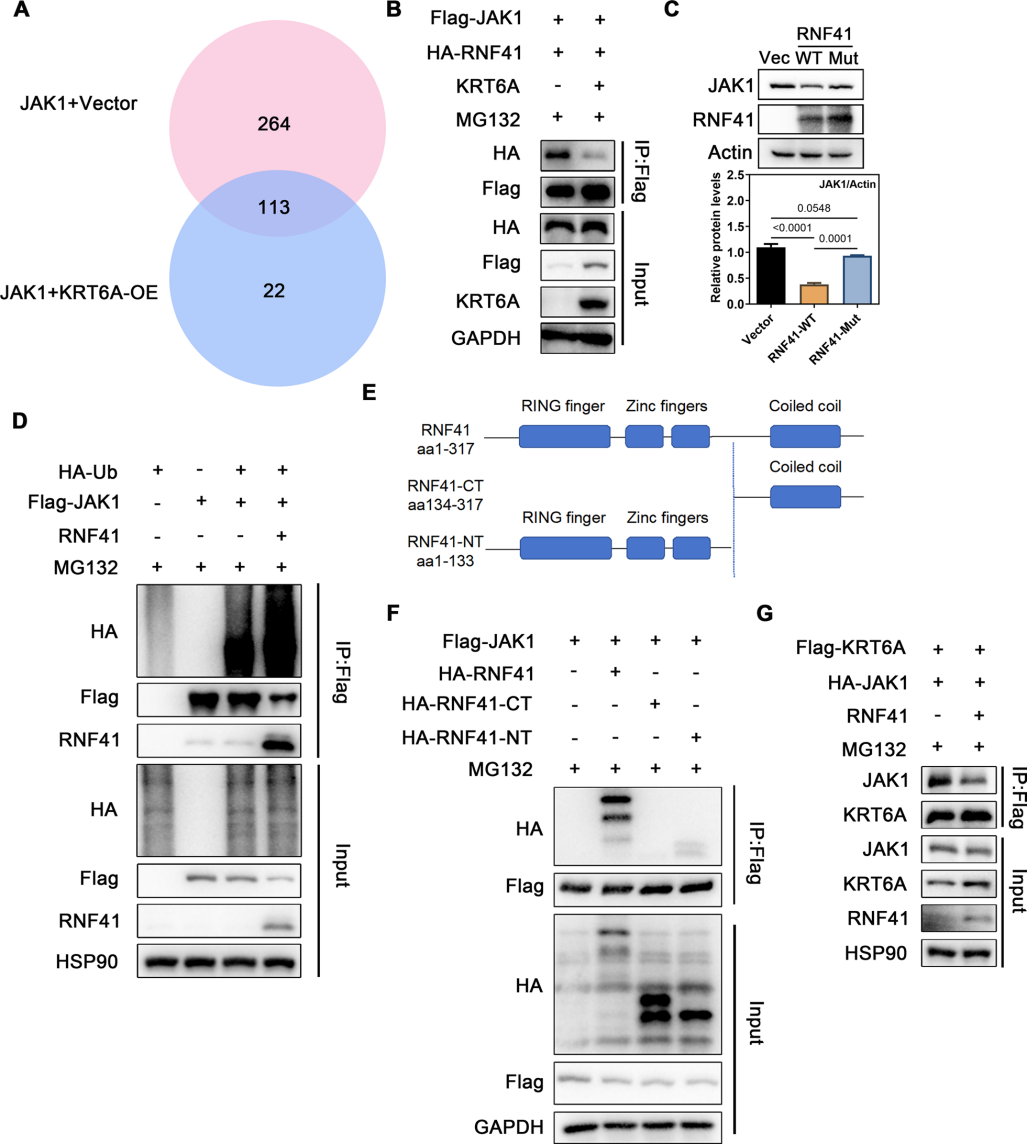
KRT6A inhibits the interaction between RNF41 and JAK1. **A** The Venn diagram of proteins interacted with JAK1 detected by IP-MS after transferring of Flag-JAK1 plasmid with or without KRT6A plasmid into HEK293T cells. **B** Flag-JAK1 and HA-RNF41 plasmids were transfected into HEK293T cells with or without KRT6A plasmid and the interaction between JAK1 and RNF41 was detected. **C** The JAK1 expression was detected in RNF41-overexpressed and enzymatic-null RNF41-expressed HEK293T cells. **D** The ubiquitination of JAK1 with or without RNF41 overexpression. **E** Schematic representation of RNF41 and its truncated forms. **F** Flag-JAK1, full-length HA-RNF41, or truncated mutants of RNF41 were coexpressed in HEK293T cells for co-IP assay with anti-Flag beads. **G** Flag-KRT6A and HA-JAK1 plasmids were transfected into HEK293T cells with or without RNF41 plasmid for co-IP assay with anti-Flag beads. All experiments were performed in three independent biological replicates

To investigate how KRT6A regulates JAK1 ubiquitination, we performed immunoprecipitation-mass spectrometry (IP-MS) to identify proteins interacting with JAK1. The results revealed that three ubiquitination-related proteins—RNF41, OBI1, and TRIM21—bound to JAK1 in the absence of KRT6A (Fig. 6A). To validate these interactions, we co-transfected JAK1 and KRT6A plasmids along with RNF41, OBI1, or TRIM21 in HEK293T cells and performed exogenous Co-IP assays. The results showed that only RNF41 interacted with JAK1, and this interaction was significantly reduced upon KRT6A overexpression (Fig. 6B and Supplementary Fig. 8A, B). Further experiments demonstrated that RNF41 enhances JAK1 ubiquitination and reduces its protein levels (Fig. 6C, D), confirming its role as a key E3 ubiquitin ligase. Furthermore, our additional experiments showed that RNF41 overexpression led to a decrease in total JAK1 protein levels without significantly altering the phosphorylation level of JAK1 (p-JAK1) (Supplementary Fig. 10C). These results suggest that RNF41-mediated JAK1 degradation occurs independently of JAK1 phosphorylation status. Structurally, RNF41 consists of an N-terminal domain (RNF41-NT, residues 1–133) that recruits E2 ubiquitin-conjugating enzymes and a C-terminal domain (RNF41-CT, residues 134–317) that binds substrates [32]. To determine which RNF41 region interacts with JAK1, we generated HA-tagged RNF41 deletion mutants and co-transfected them with Flag-tagged JAK1 in HEK293T cells (Fig. 6E). Interestingly, JAK1 interacted with the RNF41-NT mutant, suggesting that this region may mediate JAK1 ubiquitination (Fig. 6F). To confirm RNF41’s role in JAK1 degradation, we constructed a catalytically inactive RNF41 mutant. This mutant failed to promote JAK1 degradation (Fig. 6C), confirming that RNF41-dependent JAK1 downregulation requires its enzymatic activity. To further explore the relationship between KRT6A, JAK1, and RNF41, we first used confocal microscopy, which confirmed partial co-localization of KRT6A and JAK1 in the cytoplasm and perinuclear region (Supplementary Fig. 10C). To further clarify the binding position between JAK1 and KRT6A, we utilized AlphaFold to predict potential interaction regions within KRT6A. Our analysis identified amino acid sequence 315–319 as a potential JAK1-binding region (Supplementary Fig. 10D). To experimentally validate this prediction, we performed site-directed mutagenesis and found that mutations in this region significantly reduced the interaction between KRT6A and JAK1 (Supplementary Fig. 10E). Additionally, co-immunoprecipitation (co-IP) assays demonstrated that RNF41 may compete with KRT6A for JAK1 binding (Fig. 6G).

Taken together, these findings establish that KRT6A competitively binds to JAK1, thereby preventing RNF41-mediated ubiquitination and degradation of JAK1. This mechanism highlights a critical role of KRT6A in regulating JAK1 stability and STAT3 activation in inflammatory skin diseases.

## Discussion

Rosacea and psoriasis are common inflammatory skin diseases that significantly impact patients' physical and mental health due to their high prevalence and distressing skin manifestations [33, 34]. Both conditions involve skin barrier disruption and immune activation, suggesting a potential shared molecular basis. Keratins, key components of the epidermal barrier, play a role in skin inflammation [35]; however, studies on KRT6A remain limited. In this study, we found that KRT6A expression was upregulated in the epidermis of both rosacea and psoriasis. Moreover, KRT6A knockdown alleviated, while its overexpression exacerbated, rosacea-like and psoriasis-like phenotypes in mice. Mechanistically, KRT6A promotes JAK1-STAT3-mediated cytokine production in keratinocytes by restricting the interaction between RNF41 and JAK1, thereby inhibiting JAK1 ubiquitination and degradation.

In this study, we observed an increase in KRT6A expression in rosacea and found a significant positive correlation between KRT6A and IGA, a broad indicator of overall inflammatory status, suggesting a strong association between KRT6A and skin inflammation. This finding aligns with previous studies highlighting the critical role of keratins in inflammatory responses and cytokine regulation [35]. Although the correlation between KRT6A and CEA (an indicator of vascular-related erythema) was not statistically significant, we observed a positive trend, suggesting a potential role for KRT6A in vascular alterations, such as vasodilation or vascular remodeling, in rosacea. This is consistent with our finding that KRT6A promotes increased vascularization in the skin (Fig. 3H). A possible explanation is that while KRT6A contributes to angiogenesis, persistent erythema in rosacea may involve additional mechanisms beyond its direct influence.

Skin barrier alarmins, including KRT6, KRT16, and KRT17, contribute to the skin barrier's composition and are rapidly induced in stressed keratinocytes [17]. Studies have shown that KRT17 regulates cytokine expression in vivo in a cell-autonomous manner, influencing the production of CXCL9, CXCL10, and CXCL11. Furthermore, KRT17 inhibition has been found to alleviate inflammation in IMQ-induced psoriasis-like dermatitis [36, 37]. However, the loss of certain keratins, such as KRT1, KRT2, and KRT5, has been associated with skin barrier defects and the upregulation of inflammatory factors [38–40]. These findings suggest that skin barrier disruption, characterized by abnormal keratin expression, plays a critical role in skin inflammation. The differential effects of keratins on inflammation may stem from their distinct functional properties. While some keratins share broad pathological roles, such as contributing to barrier dysfunction, their molecular mechanisms are not universally conserved. Beyond barrier function, keratins also play essential roles in other biological processes. Our findings (Fig. 1D, E) confirm that KRT6A is expressed in both the epidermis and hair follicles, consistent with previous studies. KRT6A marks interfollicular epidermis and outer root sheath cells and has been used in KRT6A-Cre mice to study hair follicle biology [41]. Additionally, keratins promote hair growth by inducing dermal papilla condensation and P-cadherin-expressing hair germ formation [42], suggesting that KRT6A may contribute to hair follicle regeneration through similar mechanisms. Future research should focus on mapping keratin subtype-specific interactomes and signaling landscapes to further elucidate their distinct and overlapping roles in skin homeostasis and inflammation.

The JAK-STAT pathway, a classical inflammation-related signaling cascade, regulates various cytokines, including IL1B, IL6, TNFα, CCL20, CXCL1, and CXCL11 [43–48]. Upon cytokine-receptor engagement, receptor-associated JAKs are activated, leading to the phosphorylation and activation of STATs, which subsequently regulate downstream gene expression. Activation of the JAK-STAT pathway downregulates skin barrier-related proteins in keratinocytes, such as KRT1, KRT6, KRT10, KRT16, KRT17, CLDN1, FLG, and LOR, thereby impairing skin barrier function [49–52]. Interestingly, KRT17 has also been shown to influence STAT3 activation [53], suggesting a complex interplay between skin barrier-associated proteins and JAK-STAT signaling in maintaining barrier integrity, consistent with our findings. Moreover, elevated STAT3 levels have been detected in rosacea and psoriasis, and JAK inhibitors have been shown to effectively alleviate clinical symptoms [54, 55]. In light of our results, the therapeutic benefits of JAK-STAT pathway inhibition may, at least in part, stem from its regulatory effects on the skin barrier.

Protein regulation is a highly intricate process, with the ubiquitin–proteasome system serving as one of its key components [56]. RNF41, an E3 ubiquitin ligase, has been reported to influence JAK2 and p-STAT3 levels; however, no direct interaction between RNF41 and JAK2 has been demonstrated [57]. In this study, we identified a direct interaction between RNF41 and JAK1, accompanied by a reduction in JAK1 protein levels. Furthermore, our additional experiments showed that RNF41 overexpression decreased total JAK1 protein levels without significantly altering its phosphorylation status. Although these findings suggest that RNF41-mediated JAK1 degradation occurs independently of phosphorylation, we acknowledge that this observation alone may not fully exclude a regulatory role of phosphorylation. Definitive confirmation would require future studies utilizing phosphorylation-deficient JAK1 mutants or specific JAK1 phosphorylation inhibitors to precisely dissect the contribution of phosphorylation to JAK1 ubiquitination and degradation. Protein–protein competition is known to significantly impact downstream cellular functions and biological processes [58]. Our findings further suggest that KRT6A competes with RNF41 for JAK1 binding, thereby attenuating RNF41-mediated JAK1 ubiquitination. Nevertheless, the precise regulatory mechanism by which KRT6A modulates the RNF41-JAK1 interaction remains to be fully elucidated.

Moreover, our findings suggest that TNF-α indirectly promotes JAK1-STAT3 activation in keratinocytes through the upregulation of KRT6A. Upon TNF-α stimulation, KRT6A expression is markedly increased, which in turn inhibits RNF41-mediated ubiquitination and degradation of JAK1, thereby stabilizing JAK1 protein levels and facilitating STAT3 phosphorylation. This indirect regulatory cascade is further supported by the observation that inhibition of TNF-α signaling effectively suppresses KRT6A upregulation under external stress stimuli, such as heat shock and UVB irradiation. These results highlight a novel TNF-α–KRT6A–RNF41–JAK1 axis that contributes to inflammatory signaling amplification in keratinocytes.

## Conclusions

We demonstrated that KRT6A upregulation in keratinocytes promotes skin inflammation through RNF41-JAK1-STAT3 axis-mediated proinflammatory signaling. These findings identify KRT6A as a potential therapeutic target for inflammatory skin diseases associated with epidermal barrier dysfunction.

## Supplementary Information

Supplementary Material 1.

## Data Availability

All data needed to assess the conclusions in this study are provided in the manuscript and/or the Supplementary Materials. Sequencing data for the mouse model have been deposited in the genome sequence archive under accession number CRA012759 (https://bigd.big.ac.cn/gsa/browse). Sequencing data for the skin lesions of rosacea patients used in this study were obtained from the genome sequence archive under accession number HRA000378 (http://bigd.big.ac.cn/gsa-human/).

## Abbreviations

KRT6A: Keratin 6A
JAK1: Janus kinase 1
STAT3: Signal transduction and activator of transcription 3
RNF41: Ring finger protein41
IMQ: Imiquimod
AD: Atopic dermatitis
Th1: Type 1 helper T cells
shRNA: Short hairpin RNA
AAV: Adeno-associated virus
Scr: Scrambled

## References

[1] Medgyesi B, Dajnoki Z, Béke G, Gáspár K, Szabó IL, Janka EA, et al. Rosacea is characterized by a profoundly diminished skin barrier. J Invest Dermatol. 2020;140(10):1938-50.e5. 32199994 10.1016/j.jid.2020.02.025

[2] Montero-Vilchez T, Segura-Fernández-Nogueras MV, Pérez-Rodríguez I, Soler-Gongora M, Martinez-Lopez A, Fernández-González A. Skin barrier function in psoriasis and atopic dermatitis: transepidermal water loss and temperature as useful tools to assess disease severity. J Clin Med. 2021. 10.3390/jcm10020359. 33477944 10.3390/jcm10020359 PMC7833436

[3] Gether L, Overgaard LK, Egeberg A. Incidence and prevalence of rosacea: a systematic review and meta-analysis. Br J Dermatol. 2018;179(2):282–9. 29478264 10.1111/bjd.16481

[4] Parisi R, Iskandar IYK, Kontopantelis E, Augustin M, Griffiths CEM, Ashcroft DM. National, regional, and worldwide epidemiology of psoriasis: systematic analysis and modelling study. BMJ (Clinical Research ed). 2020;369: m1590. 32467098 10.1136/bmj.m1590 PMC7254147

[5] Darlenski R, Kazandjieva J, Tsankov N, Fluhr JW. Acute irritant threshold correlates with barrier function, skin hydration and contact hypersensitivity in atopic dermatitis and rosacea. Exp Dermatol. 2013;22(11):752–3. 24112695 10.1111/exd.12251

[6] Leigh IM, Navsaria H, Purkis PE, McKay IA, Bowden PE, Riddle PN. Keratins (K16 and K17) as markers of keratinocyte hyperproliferation in psoriasis in vivo and in vitro. Br J Dermatol. 1995;133(4):501–11. 7577575 10.1111/j.1365-2133.1995.tb02696.x

[7] Rendon A, Schäkel K. Psoriasis pathogenesis and treatment. Int J Mol Sci. 2019. 10.3390/ijms20061475. 30909615 10.3390/ijms20061475 PMC6471628

[8] Herster F, Bittner Z, Archer NK, Dickhöfer S, Eisel D, Eigenbrod T, et al. Neutrophil extracellular trap-associated RNA and LL37 enable self-amplifying inflammation in psoriasis. Nat Commun. 2020;11(1):105. 31913271 10.1038/s41467-019-13756-4 PMC6949246

[9] Mylonas A, Hawerkamp HC, Wang Y, Chen J, Messina F, Demaria O, et al. Type I IFNs link skin-associated dysbiotic commensal bacteria to pathogenic inflammation and angiogenesis in rosacea. JCI Insight. 2023. 10.1172/jci.insight.151846. 36633910 10.1172/jci.insight.151846 PMC9977509

[10] De Benedetto A, Rafaels NM, McGirt LY, Ivanov AI, Georas SN, Cheadle C, et al. Tiht junction defects in patients with atopic dermatitis. J Allergy Clin Immunol. 2011;127(3):773-86.e1-7. 21163515 10.1016/j.jaci.2010.10.018 PMC3049863

[11] Knox SM, Erwin EA, Mosser-Goldfarb JL, Scherzer R. Sensitization patterns among patients with atopic dermatitis evaluated in a large tertiary care pediatric center. Ann Allergy Asthma Immunol. 2017;118(5):645–7. 28372896 10.1016/j.anai.2017.03.006

[12] Spergel JM, Mizoguchi E, Brewer JP, Martin TR, Bhan AK, Geha RS. Epicutaneous sensitization with protein antigen induces localized allergic dermatitis and hyperresponsiveness to methacholine after single exposure to aerosolized antigen in mice. J Clin Investig. 1998;101(8):1614–22. 9541491 10.1172/JCI1647 PMC508742

[13] Elias PM. Epidermal lipids, barrier function, and desquamation. J Invest Dermatol. 1983;80(1 Suppl):44s-s49. 20479733 10.1038/jid.1983.12

[14] Ekanayake-Mudiyanselage S, Aschauer H, Schmook FP, Jensen JM, Meingassner JG, Proksch E. Expression of epidermal keratins and the cornified envelope protein involucrin is influenced by permeability barrier disruption. J Invest Dermatol. 1998;111(3):517–23. 9740250 10.1046/j.1523-1747.1998.00318.x

[15] Oppenheim JJ, Yang D. Alarmins: chemotactic activators of immune responses. Curr Opin Immunol. 2005;17(4):359–65. 15955682 10.1016/j.coi.2005.06.002

[16] Takahashi K, Paladini RD, Coulombe PA. Cloning and characterization of multiple human genes and cDNAs encoding highly related type II keratin 6 isoforms. J Biol Chem. 1995;270(31):18581–92. 7543104 10.1074/jbc.270.31.18581

[17] DePianto D, Coulombe PA. Intermediate filaments and tissue repair. Exp Cell Res. 2004;301(1):68–76. 15501447 10.1016/j.yexcr.2004.08.007

[18] Yu G, Wang LG, Han Y, He QY. clusterProfiler: an R package for comparing biological themes among gene clusters. OMICS. 2012;16(5):284–7. 22455463 10.1089/omi.2011.0118 PMC3339379

[19] Yuan X, Li J, Li Y, Deng Z, Zhou L, Long J, et al. Artemisinin, a potential option to inhibit inflammation and angiogenesis in rosacea. Biomed Pharmacotherapy. 2019;117: 109181. 10.1016/j.biopha.2019.109181 31387196

[20] Deng Z, Chen M, Xie H, Jian D, Xu S, Peng Q. Claudin reduction may relate to an impaired skin barrier in rosacea. J Dermatol. 2019;46(4):314–21. 30714633 10.1111/1346-8138.14792

[21] UniProt Consortium. UniProt: the universal protein knowledgebase in 2023. Nucleic Acids Res. 2023;51(D1):D523–31. 36408920 10.1093/nar/gkac1052 PMC9825514

[22] Mirdita M, Schütze K, Moriwaki Y, Heo L, Ovchinnikov S, Steinegger M. ColabFold: making protein folding accessible to all. Nat Methods. 2022;19(6):679–82. 35637307 10.1038/s41592-022-01488-1 PMC9184281

[23] Tunyasuvunakool K, Adler J, Wu Z, Green T, Zielinski M, Žídek A, Bridgland A, et al. Highly accurate protein structure prediction for the human proteome. Nature. 2021;596(7873):590–6. 34293799 10.1038/s41586-021-03828-1 PMC8387240

[24] Yamasaki K, Di Nardo A, Bardan A, Murakami M, Ohtake T, Coda A, et al. Increased serine protease activity and cathelicidin promotes skin inflammation in rosacea. Nat Med. 2007;13(8):975–80. 17676051 10.1038/nm1616

[25] Holmes AD, Steinhoff M. Integrative concepts of rosacea pathophysiology, clinical presentation and new therapeutics. Exp Dermatol. 2017;26(8):659–67. 27376863 10.1111/exd.13143

[26] Zhang Y, Li Y, Zhou L, Yuan X, Wang Y, Deng Q, et al. Nav1.8 in keratinocytes contributes to ROS-mediated inflammation in inflammatory skin diseases. Redox Biol. 2022;55: 102427. 35952475 10.1016/j.redox.2022.102427 PMC9372634

[27] Wladis EJ, Swamy S, Herrmann A, Yang J, Carlson JA, Adam AP. Activation of p38 and Erk mitogen-activated protein kinases signaling in ocular Rosacea. Invest Ophthalmol Vis Sci. 2017;58(2):843–8. 28170535 10.1167/iovs.16-20275

[28] Wang Y, Wang B, Huang Y, Li Y, Yan S, Xie H, et al. Multi-transcriptomic analysis and experimental validation implicate a central role of STAT3 in skin barrier dysfunction induced aggravation of Rosacea. J Inflamm Res. 2022;15:2141–56. 35392024 10.2147/JIR.S356551 PMC8980297

[29] Jiang X, Fang L, Wu H, Mei X, He F, Ding P, et al. TLR2 regulates allergic airway inflammation and autophagy through PI3K/Akt signaling pathway. Inflammation. 2017;40(4):1382–92. 28493079 10.1007/s10753-017-0581-x

[30] Kim H, Frederick DT, Levesque MP, Cooper ZA, Feng Y, Krepler C, et al. Downregulation of the ubiquitin ligase RNF125 underlies resistance of melanoma cells to BRAF inhibitors via JAK1 deregulation. Cell Rep. 2015;11(9):1458–73. 26027934 10.1016/j.celrep.2015.04.049 PMC4681438

[31] Li M, Xu Y, Liang J, Lin H, Qi X, Li F, et al. USP22 deficiency in melanoma mediates resistance to T cells through IFNγ-JAK1-STAT1 signal axis. Mol Therapy J Am Soc Gene Therapy. 2021;29(6):2108–20. 10.1016/j.ymthe.2021.02.018 PMC8178440 33601053

[32] Qiu XB, Goldberg AL. Nrdp1/FLRF is a ubiquitin ligase promoting ubiquitination and degradation of the epidermal growth factor receptor family member, ErbB3. Proc Natl Acad Sci USA. 2002;99(23):14843–8. 12411582 10.1073/pnas.232580999 PMC137506

[33] Cortés H, Rojas-Márquez M, Del Prado-Audelo ML, Reyes-Hernández OD, González-Del CM. Alterations in mental health and quality of life in patients with skin disorders: a narrative review. Int J Dermatol. 2022;61(7):783–91. 34403497 10.1111/ijd.15852

[34] Singam V, Rastogi S, Patel KR, Lee HH, Silverberg JI. The mental health burden in acne vulgaris and rosacea: an analysis of the US National Inpatient Sample. Clin Exp Dermatol. 2019;44(7):766–72. 30706514 10.1111/ced.13919

[35] Lessard JC, Piña-Paz S, Rotty JD, Hickerson RP, Kaspar RL, Balmain A, et al. Keratin 16 regulates innate immunity in response to epidermal barrier breach. Proc Natl Acad Sci USA. 2013;110(48):19537–42. 24218583 10.1073/pnas.1309576110 PMC3845144

[36] Depianto D, Kerns ML, Dlugosz AA, Coulombe PA. Keratin 17 promotes epithelial proliferation and tumor growth by polarizing the immune response in skin. Nat Genet. 2010;42(10):910–4. 20871598 10.1038/ng.665 PMC2947596

[37] Xiao CY, Zhu ZL, Zhang C, Fu M, Qiao HJ, Wang G, et al. Small interfering RNA targeting of keratin 17 reduces inflammation in imiquimod-induced psoriasis-like dermatitis. Chin Med J. 2020;133(24):2910–8. 33237695 10.1097/CM9.0000000000001197 PMC7752698

[38] Roth W, Kumar V, Beer HD, Richter M, Wohlenberg C, Reuter U, et al. Keratin 1 maintains skin integrity and participates in an inflammatory network in skin through interleukin-18. J Cell Sci. 2012;125(Pt 22):5269–79. 23132931 10.1242/jcs.116574

[39] Fischer H, Langbein L, Reichelt J, Praetzel-Wunder S, Buchberger M, Ghannadan M, et al. Loss of keratin K2 expression causes aberrant aggregation of K10, hyperkeratosis, and inflammation. J Invest Dermatol. 2014;134(10):2579–88. 24751727 10.1038/jid.2014.197

[40] Lu H, Chen J, Planko L, Zigrino P, Klein-Hitpass L, Magin TM. Induction of inflammatory cytokines by a keratin mutation and their repression by a small molecule in a mouse model for EBS. J Invest Dermatol. 2007;127(12):2781–9. 17581617 10.1038/sj.jid.5700918

[41] Smyth I, Ellis T, Hetherington R, Riley E, Narang M, Mahony D, et al. Krt6a-Cre transgenic mice direct LoxP-mediated recombination to the companion cell layer of the hair follicle and following induction by retinoic acid to the interfollicular epidermis. J Invest Dermatol. 2004;122(1):232–4. 14962113 10.1046/j.0022-202X.2003.22122.x

[42] An SY, Kim HS, Kim SY, Van SY, Kim HJ, Lee JH, et al. Keratin-mediated hair growth and its underlying biological mechanism. Commun Biol. 2022;5(1):1270. 36402892 10.1038/s42003-022-04232-9 PMC9675858

[43] Tian DS, Peng J. Chemokine CCL2-CCR2 signaling induces neuronal cell death via STAT3 activation and IL-1β production after status epilepticus. J Neurosci. 2017;37(33):7878–92. 28716963 10.1523/JNEUROSCI.0315-17.2017 PMC5559763

[44] Lieblein JC, Ball S, Hutzen B, Sasser AK, Lin HJ, Huang TH, et al. STAT3 can be activated through paracrine signaling in breast epithelial cells. BMC Cancer. 2008;8:302. 18939993 10.1186/1471-2407-8-302 PMC2582243

[45] Yun JH, Lee DH, Jeong HS, Kim SH, Ye SK, Cho CH. STAT3 activation in microglia increases pericyte apoptosis in diabetic retinas through TNF-ɑ/AKT/p70S6 kinase signaling. Biochem Biophys Res Commun. 2022;613:133–9. 35561580 10.1016/j.bbrc.2022.05.004

[46] Chen C, Yang Z, Yin X, Huang S, Yan J, Sun Q. CircEIF5 contributes to hyperproliferation and inflammation of keratinocytes in psoriasis via p-NFκB and p-STAT3 signalling pathway. Exp Dermatol. 2022;31(8):1145–53. 35288970 10.1111/exd.14565

[47] Lee MJ, Lee J, Kang SK, Wirth D, Yoo SM, Park C, et al. CXCL1 confers a survival advantage in Kaposi’s sarcoma-associated herpesvirus-infected human endothelial cells through STAT3 phosphorylation. J Med Virol. 2023. 10.1002/jmv.28020. 35869037 10.1002/jmv.28020

[48] Yang CH, Wei L, Pfeffer SR, Du Z, Murti A, Valentine WJ, et al. Identification of CXCL11 as a STAT3-dependent gene induced by IFN. J Immunol (Baltimore, Md: 1950). 2007;178(2):986–92. 10.4049/jimmunol.178.2.986 17202361

[49] Qiao P, Zhi D, Yu C, Zhang C, Wu K, Fang H, et al. Activation of the C3a anaphylatoxin receptor inhibits keratinocyte proliferation by regulating keratin 6, keratin 16, and keratin 17 in psoriasis. FASEB J. 2022;36(5): e22322. 35429062 10.1096/fj.202101458R

[50] Dai X, Shiraishi K, Muto J, Utsunomiya R, Mori H, Murakami M, et al. Nuclear IL-33 plays an important role in IL-31-mediated downregulation of FLG, Keratin 1, and Keratin 10 by regulating signal transducer and activator of transcription 3 activation in human keratinocytes. J Invest Dermatol. 2022;142(1):136-44.e3. 34293350 10.1016/j.jid.2021.05.033

[51] Ryu WI, Lee H, Bae HC, Jeon J, Ryu HJ, Kim J, et al. IL-33 down-regulates CLDN1 expression through the ERK/STAT3 pathway in keratinocytes. J Dermatol Sci. 2018;90(3):313–22. 29534857 10.1016/j.jdermsci.2018.02.017

[52] Amano W, Nakajima S, Kunugi H, Numata Y, Kitoh A, Egawa G, et al. The Janus kinase inhibitor JTE-052 improves skin barrier function through suppressing signal transducer and activator of transcription 3 signaling. J Allergy Clin Immunol. 2015;136(3):667-77.e7. 26115905 10.1016/j.jaci.2015.03.051

[53] Yang L, Jin L, Ke Y, Fan X, Zhang T, Zhang C, et al. E3 ligase Trim21 ubiquitylates and stabilizes Keratin 17 to induce STAT3 activation in psoriasis. J Invest Dermatol. 2018;138(12):2568–77. 29859926 10.1016/j.jid.2018.05.016

[54] Papp KA, Menter MA, Abe M, Elewski B, Feldman SR, Gottlieb AB, et al. Tofacitinib, an oral Janus kinase inhibitor, for the treatment of chronic plaque psoriasis: results from two randomized, placebo-controlled, phase III trials. Br J Dermatol. 2015;173(4):949–61. 26149717 10.1111/bjd.14018

[55] Sun YH, Man XY. Tofacitinib for the treatment of erythematotelangiectatic and papulopustular rosacea: a retrospective case series. Dermatol Ther. 2022;35(11): e15848. 36175135 10.1111/dth.15848

[56] Pohl C, Dikic I. Cellular quality control by the ubiquitin-proteasome system and autophagy. Science (New York, NY). 2019;366(6467):818–22. 10.1126/science.aax3769 31727826

[57] Wauman J, De Ceuninck L, Vanderroost N, Lievens S, Tavernier J. RNF41 (Nrdp1) controls type 1 cytokine receptor degradation and ectodomain shedding. J Cell Sci. 2011;124(Pt 6):921–32. 21378310 10.1242/jcs.078055 PMC3115735

[58] Xu Y, Liang C. Possible mechanism of GATA4 inhibiting myocardin activity during cardiac hypertrophy. J Cell Biochem. 2019;120(6):9047–55. 30582211 10.1002/jcb.28178

