# Supplementary material for: Keratin 6A promotes skin inflammation through JAK1-STAT3 activation in keratinocytes
Source: J Biomed Sci. 2025 May 9;32:47. doi: 10.1186/s12929-025-01143-9 (PMC12065298; doi:10.1186/s12929-025-01143-9)
Supplement: Supplementary file 1 — Supplementary Material 1. [file 12929_2025_1143_MOESM1_ESM.pdf]

SUPPLEMENTARY MATERIALS

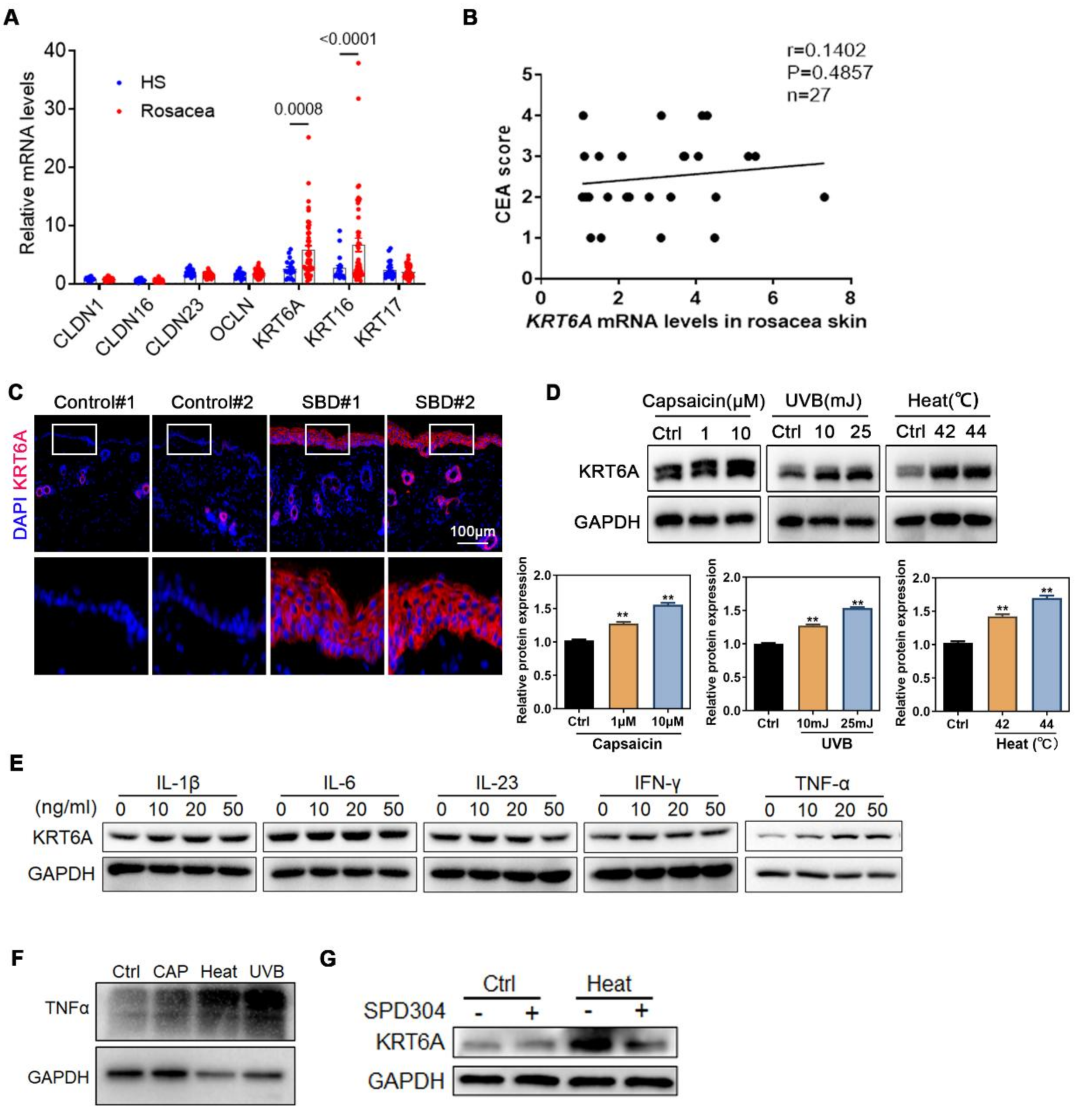

Figure S1. KRT6A is up-regulated in irritated conditions of rosacea and psoriasis.

(A) The mRNA levels of skin barrier alarms in rosacea lesions(n=16) and healthy controls(n=19). (B) Correlation analysis between KRT6A expression and CEA score in rosacea lesions. (C) Representative IF images showing the expression of KRT6A in the skin of mice with skin barrier dysfunction by tape-stripping. SBD: skin barrier dysfunction. (D) Representative western

blot images showing the expression of KRT6A in HaCaT cells treated with capsaicin, UVB, or high temperatures. Quantification of relative protein expression is shown in the bottom panel. (E) Representative western blot images showing the expression of KRT6A in HaCaT cells treated with various concentration of IL-1β, IL-6, IL-23, IFN-γ or TNF-α. (F) Representative western blot images showing the expression of TNFα in HaCaT cells treated with capsaicin, UVB, or high temperatures. (G) Representative western blot images showing the expression of KRT6A in HaCaT cells treated with high temperatures and with or without SPD304 (2μM). Two-tailed unpaired Student's t-test (D) was used. All experiments were performed in 3 independent biological replicates. Data represents the mean ± SD. *$p < 0.05$, **$p < 0.01$. Two-tailed unpaired Student's t-test (D) was used.

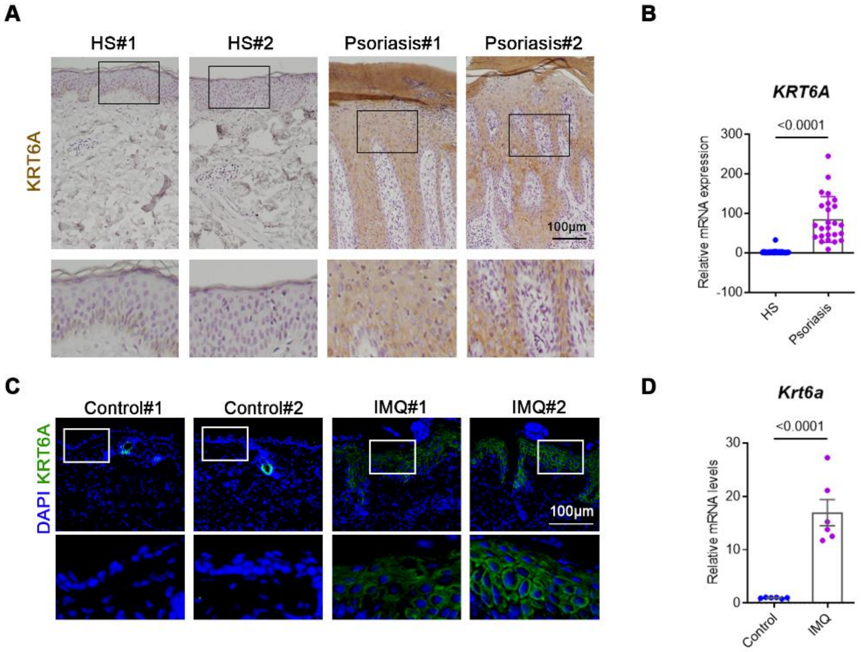

Figure S2. KRT6A is up-regulated in psoriasis.

(A) Representative IHC images showing the expression of KRT6A in psoriasis

lesions and healthy controls. (B) The mRNA levels of KRT6A in psoriasis lesions and healthy controls from the GEO database (GSE121212). IF images (C) and RT-qPCR analysis (D) showing the expression of KRT6A in the skin of IMQ-induced psoriasis-like mice and controls.

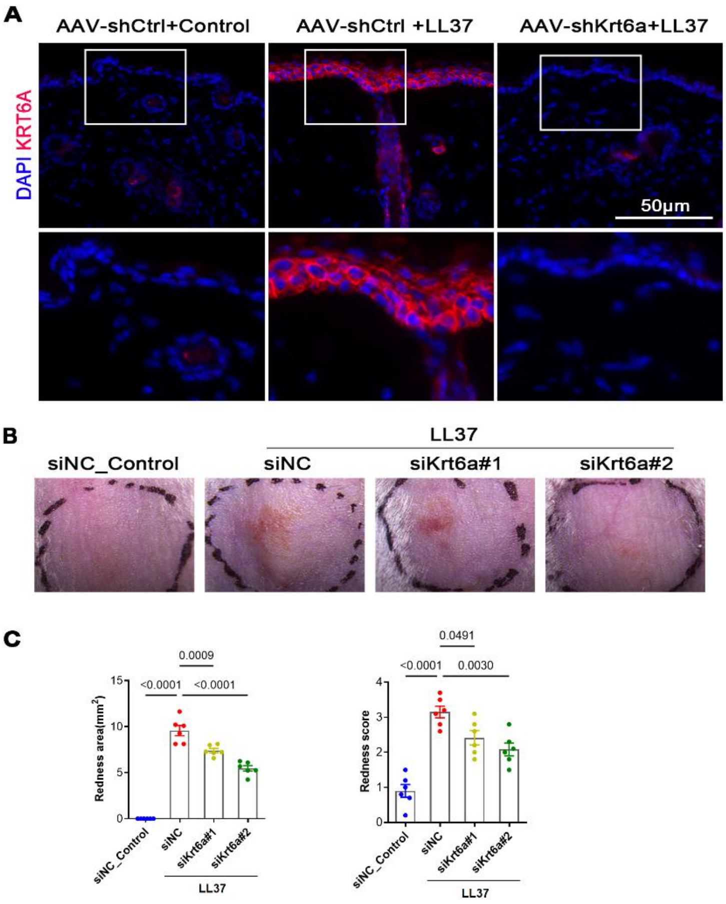

Figure S3. KRT6A knockdown relieves the development of rosacea.

(A) The knockdown efficiency of KRT6A by AAV in LL37-treated mice. (B) The back skins of the control group and siRNA-mediated knockdown of the Krt6a group treated with or without LL37 (n=6/group). Images were taken 48 hr after the first LL37 injection. (C)The redness score and redness area of the back skin of mice in (B). Data represents the mean ± SEM. The one-way ANOVA

with Bonferroni's post hoc tests was used for multiple comparisons.

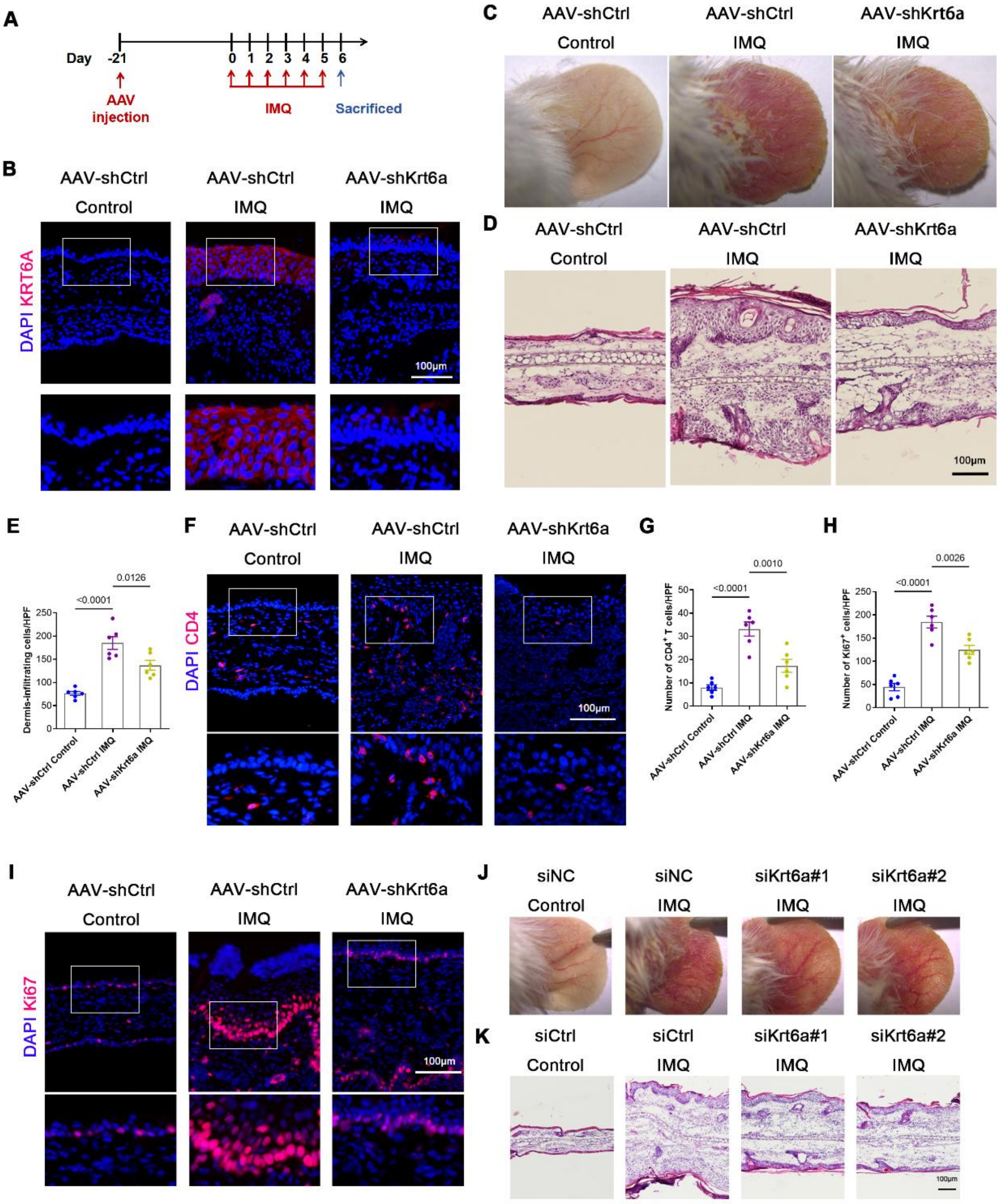

Figure S4. KRT6A knockdown relieves the development of psoriasis.

(A) Schematic diagram of AAV-shKrt6a-injected mice treated with IMQ or control. (B) The knockdown efficiency of Krt6a by AAV in IMQ-treated mice. (C) Ear skin of mice in control group and Krt6a-knockdown group treated with IMQ (n=6/group). (D) HE staining and (E) dermal infiltrating cell counts of the ear in

mice. The CD4+ T cell infiltration (F) and cell counts (G) in skin visualized by IF. The Ki67+ cells (H) and cell counts (I) in the skin visualized by IF. (J) The ear skins of the control group and siRNA-mediated knockdown of the Krt6a group treated with or without IMQ (n=6/group). (K) HE staining of the ear in mice. Data represents the mean ± SEM. The one-way ANOVA with Bonferroni's post hoc tests was used for multiple comparisons.

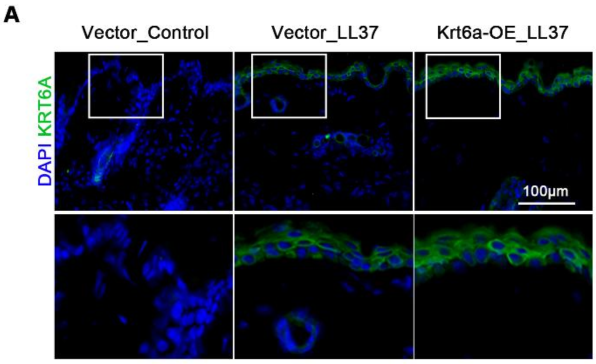

Figure S5. The efficiency of Krt6a overexpression by lentivirus in LL37-treated mice.

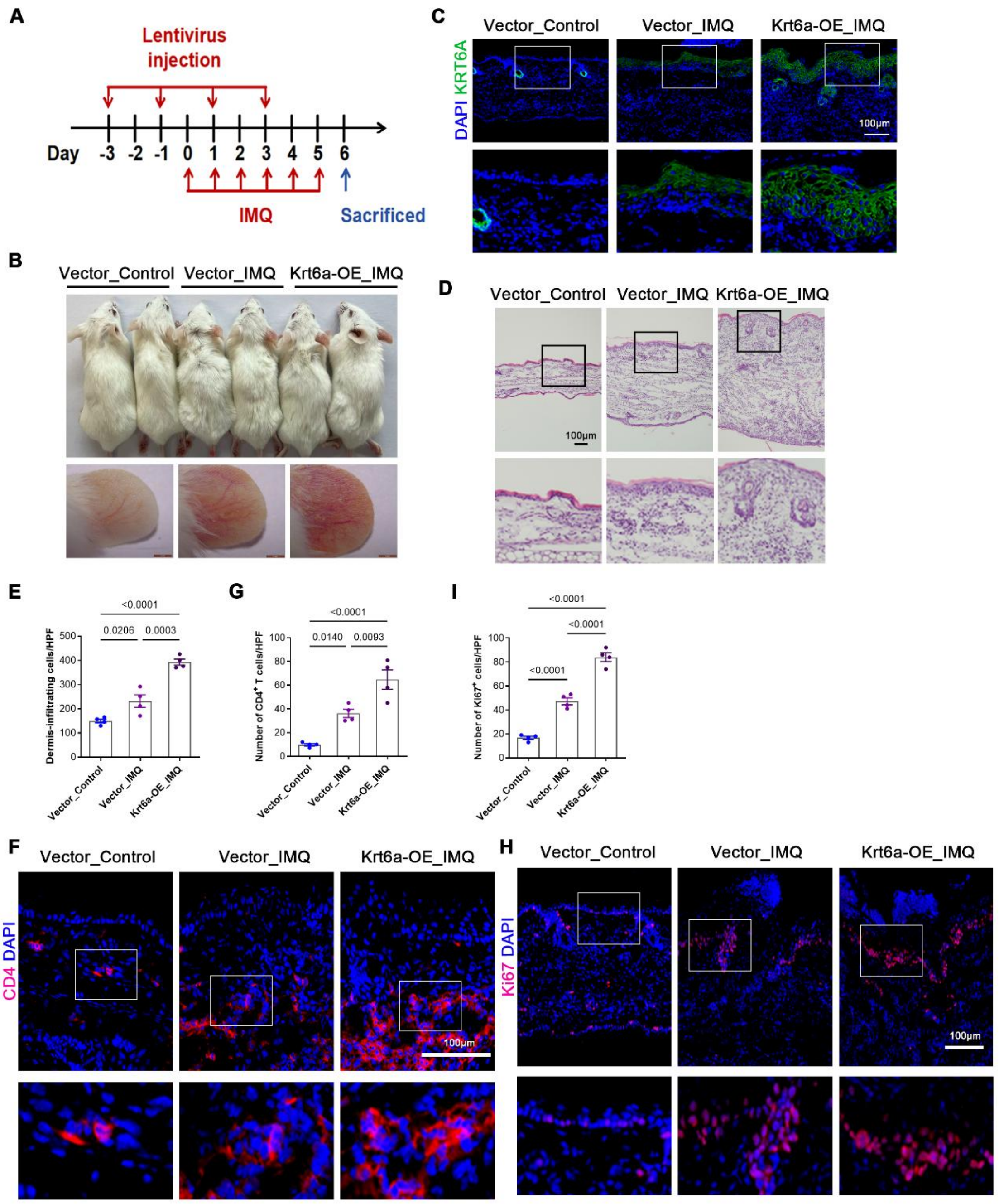

Figure S6. KRT6A promotes the development of psoriasis.

(A) Schematic diagram of lentivirus-injected mice treated with IMQ or control. (B) Ear skin of mice in the control group and Krt6a-overexpressed group treated with IMQ. (C) Krt6a overexpressed by lentivirus in IMQ-treated mice. (D) HE staining and (E) dermal infiltrating cell counts of the ear in mice. (F) The CD4+ T cell infiltration and (G) cell counts in the skin visualized by IF. (H) The

Ki67+ cells, and cell counts (I) in skin visualized by IF. Data represents the mean ± SEM. The one-way ANOVA with Bonferroni's posthoc tests was used for multiple comparisons.

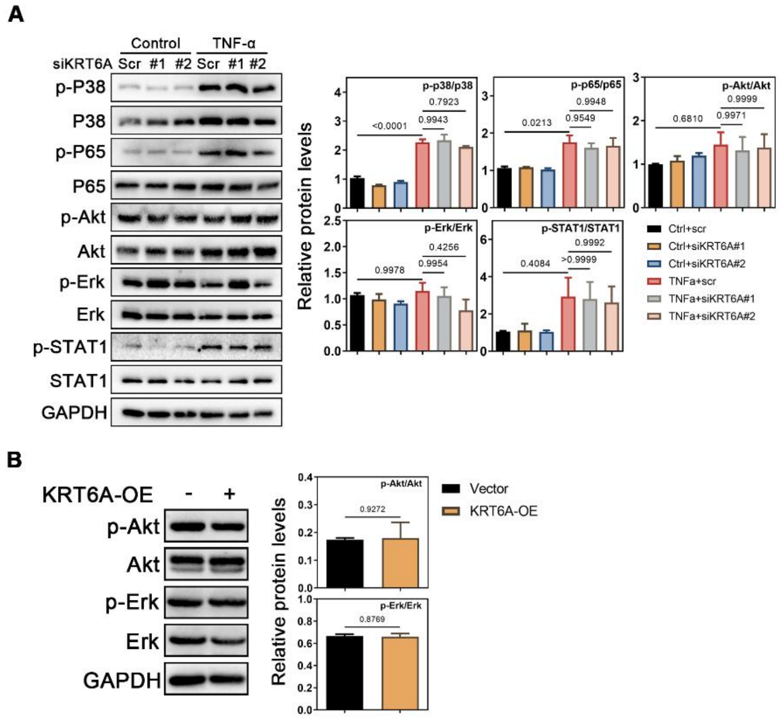

Figure S7. KRT6A affects STAT3 activation in keratinocytes.

(A)Immunoblotting of p-STAT1, STAT1, p-p65, P65, p-p38, P38, p-AKT, AKT, p-ERK or ERK in cell lysates from HaCaT cells infected with siKRT6A or Scr and stimulated with TNF-α for 2 hours. Quantification of relative protein expression is shown in the right panel. (B) Immunoblotting of p-AKT, AKT, p-ERK or ERK in cell lysates from KRT6A-overexpressed HaCaT cells. Quantification of relative protein expression is shown in the right panel. All experiments were performed in 3 independent biological replicates. Data represents the mean ± SEM. Two-tailed unpaired Student's t-test (B) or 1-way ANOVA with Bonferroni's post hoc test (A) was used.

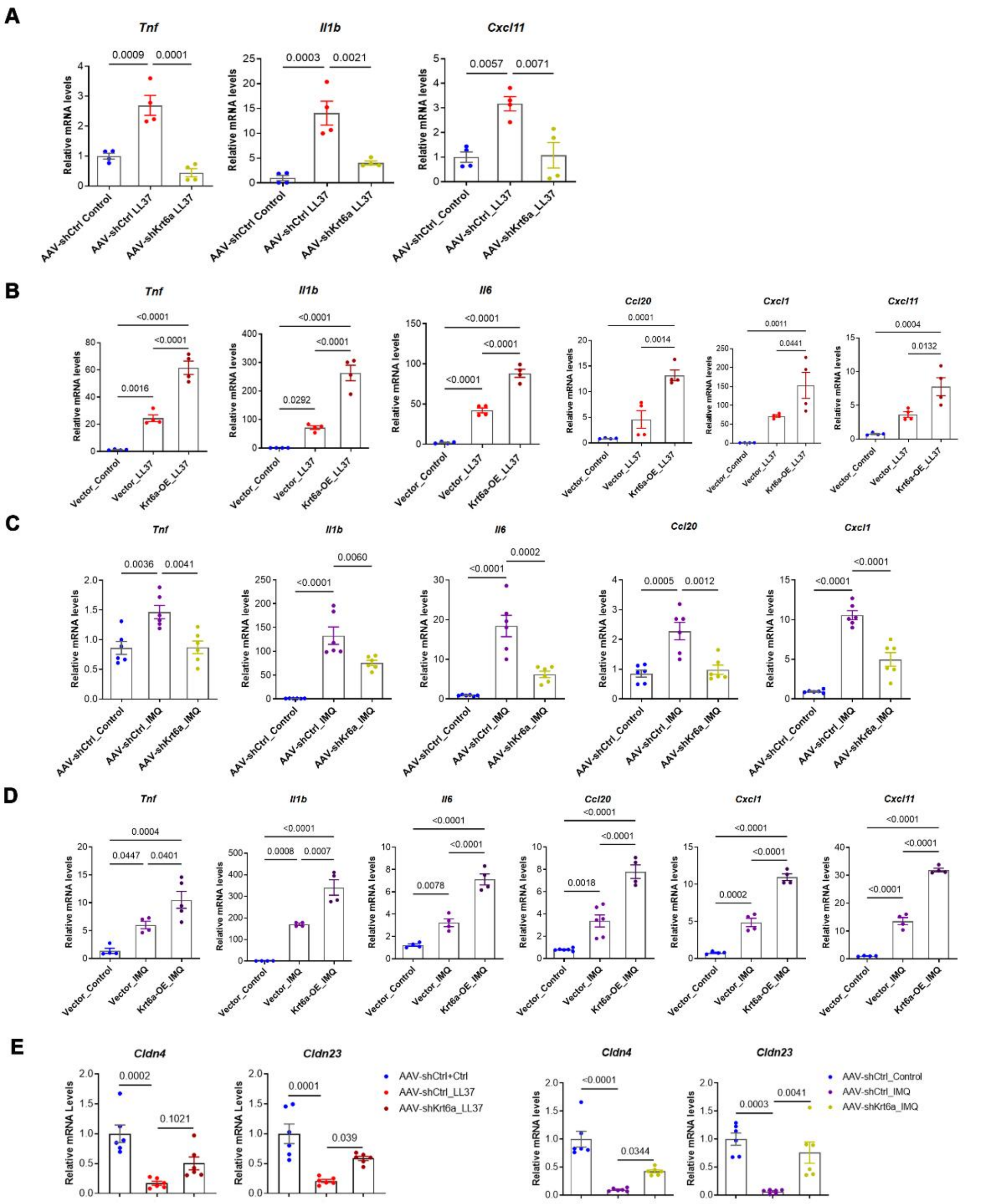

Figure S8. KRT6A affects cytokine expression in vivo.

The mRNA levels of cytokines in LL37-induced mice combined with Krt6a knockdown (A) or overexpression (B). The mRNA levels of cytokines in IMQ-induced mice combined with Krt6a knockdown (C) or overexpression (D). (E) The mRNA levels of *Cldn4* and *Cldn23* in LL37/IMQ-induced mice combined with Krt6a knockdown. Data represents the mean ± SEM. The

one-way ANOVA with Bonferroni's posthoc tests was used for multiple comparisons.

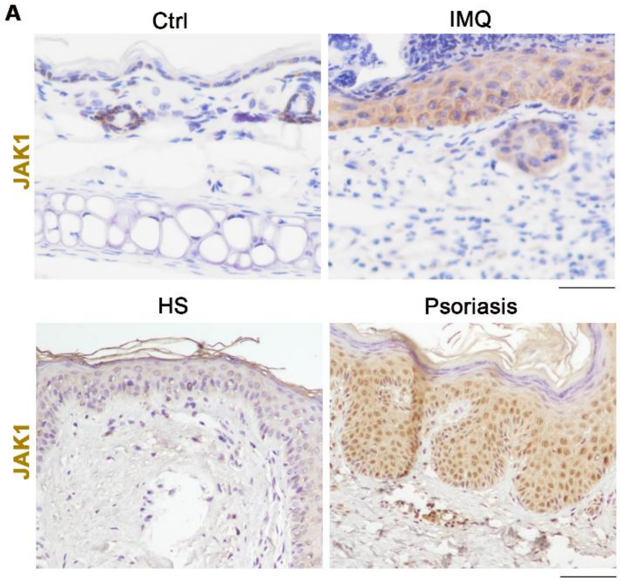

Figure S9. JAK1 expression in human and mouse models of psoriasis

Representative IHC images showing the expression of JAK1 in psoriatic lesions and healthy controls in the upper panel; Representative IHC images showing the expression of JAK1 in IMQ-induced rosacea-like mice in the bottom panel.

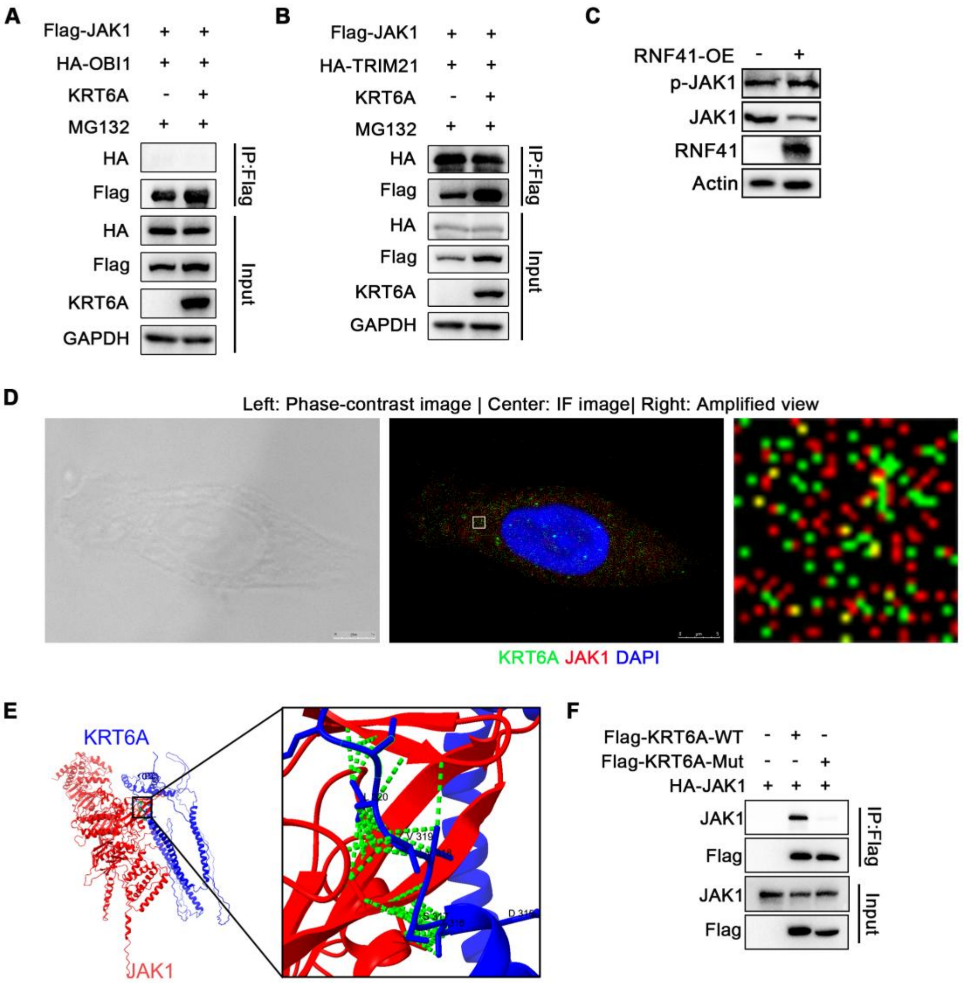

Figure S10. HA-OBI1 plasmid (A) or HA-TRIM21 plasmid (B) combined with Flag-JAK1 plasmid was transfected into HEK293T cells with or without KRT6A plasmid to detect the interaction between JAK1 and the target protein. (C) RNF41 plasmid was transfected into HaCaT cells. (D) Representative images showing the localization of KRT6A and JAK1 in HaCaT cells.Left: Corresponding phase-contrast image confirming intact cell morphology and the spatial localization of the fluorescence signals.Center: Immunofluorescence image illustrating the distribution of KRT6A and JAK1. Right: Magnified view of the region of interest, highlighting the cytoplasmic colocalization signals. Scale bar = 5 μm. (E) Diagram of the predicted

KRT6A-JAK1 binding region using AlphaFold. (F) HA-JAK1, wildtype Flag-KRT6A, or mutant (mut) of KRT6A were coexpressed in HEK293T cells for co-IP assay with anti-Flag beads.

Supplementary Table S1. The List of Primer Sequences for RT-qPCR

Gene	Species	Forward sequence	Reverse sequence
GAPDH	human	TGTTGCCATCAATGACCCCTT	CTCCACGACGTACTCAGCG
IL1B	human	AGCTACGAATCTCCGACCAC	CGTTATCCCATGTGTCGAAGAA
IL6	human	CCTGAACCTTCCAAAGATGGC	TTCACCAGGCAAGTCTCCTCA
TNFa	human	CCTCTCTCTAATCAGCCCTCTG	GAGGACCTGGGAGTAGATGAG
CCL20	human	TGCTGTACCAAGAGTTTGCTC	CGCACACAGACAACTTTTTCTTT
CXCL1	human	GCCCAAACCGAAGTCATAGCC	ATCCGCCAGCCTCTATCACA
CXCL11	human	GACGCTGTCTTTGCATAGGC	GGATTTAGGCATCGTTGTCCTTT
KRT6A	human	ATAAGTGTTGTGAACCCCCACC	GCAATTGCAAACAGCGAAGAG
Gapdh	mouse	AGGTCGGTGTGAACGGATTTG	TGTAGACCATGTAGTTGAGGTCA
Il1b	mouse	GCAACTGTTCCTGAACTCAACT	ATCTTTTGGGGTCCGTCAACT
Il6	mouse	TAGTCCTTCCTACCCCAATTTCC	TTGGTCCTTAGCCACTCCTTC
Tnfa	mouse	CTGAACTTCGGGGTGATCGG	GGCTTGTCACTCGAATTTTGAGA
Ccl20	mouse	GCCTCTCGTACATACAGACGC	CCAGTTCTGCTTTGGATCAGC
Cxcl1	mouse	CTGGGATTCACCTCAAGAACATC	CAGGGTCAAGGCAAGCCTC
Cxcl11	mouse	GGCTTCCTTATGTTCAAACAGGG	GCCGTTACTCGGGTAAATTACA
Cldn4	mouse	GTCCTGGGAATCTCCTTGGC	TCTGTGCCGTGACGATGTTG

Cldn23	mouse	CCCGACGAGTGGAACTACTTC	GGCCAGCGACGAAAAACAC

Supplementary Table S2. The List of Antibody Information

Antibodies	Source	Identifier	Dilution
Anti-human KRT6A	Sigma-Aldrich	HPA061168	WB: 1:1000<br>IHC: 1:500
Anti-human JAK1	Proteintech	66466-1-Ig	WB: 1:2000
Anti-human pSTAT3	Cell Signaling Technology	9145S	WB: 1:2000<br>IF: 1:50
Anti-human STAT3	Proteintech	10253-2-AP	WB: 1:2000
Anti-human RNF41	Proteintech	17233-1-AP	WB: 1:500
Anti-FLAG	Thermo Fisher Scientific	MA1-91878	WB: 1:1000
Anti-HA	Cell Signaling Technology	3724S	WB: 1:1000
Anti-HSP90	Proteintech	60318-1-Ig	WB: 1:5000
Anti-LAMIN B1	Proteintech	66095-1-Ig	WB: 1:1000
Anti-GAPDH	Bioworlde Technology	AP0066	WB: 1:500
Anti-TUBULIN	Bioworlde Technology	BS1482M	WB: 1:1000
Anti-mouse CD4	Thermo Fisher Scientific	14-0042-85	IF: 1:100
Anti-mouse CD31	BD Biosciences	558736	IF: 1:100
Anti-mouse CD31	Cell Signaling Technology	77699	IHC: 1:100

Anti-mouse KRT6A	Abcam	Company-designed	IHC: 1:500
Anti-mouse Ki67	Thermo Fisher Scientific	14-5698-80	IF: 1:500
Donkey anti-Rabbit, Alexa FluorTM594	Thermo Fisher Scientific	A-21207	IF: 1:500
Donkey anti-Rabbit, Alexa FluorTM488	Thermo Fisher Scientific	A-21206	IF: 1:500
Donkey anti-Rat, Alexa FluorTM594	Thermo Fisher Scientific	A-21209	IF: 1:500
Goat Anti-Rabbit IgG H&L	ZSGB-BIO	ZB-2301	WB: 1:20000
Goat Anti-Mouse IgG H&L	ZSGB-BIO	ZB-2305	WB: 1:20000
